# The Impact of the Invasive Alien Plant, *Impatiens glandulifera*, on Pollen Transfer Networks

**DOI:** 10.1371/journal.pone.0143532

**Published:** 2015-12-03

**Authors:** Carine Emer, Ian P. Vaughan, Simon Hiscock, Jane Memmott

**Affiliations:** 1 Bristol Life Sciences Building, University of Bristol, Bristol, United Kingdom; 2 Departamento de Ecologia, Universidade Estadual Paulista—UNESP, Rio Claro, Brazil; 3 School of Biosciences, Cardiff University, Cardiff, United Kingdom; 4 The University of Oxford Botanic Garden, Oxford, United Kingdom; Central China Normal University, CHINA

## Abstract

Biological invasions are a threat to the maintenance of ecological processes, including pollination. Plant-flower visitor networks are traditionally used as a surrogated for pollination at the community level, despite they do not represent the pollination process, which takes place at the stigma of plants where pollen grains are deposited. Here we investigated whether the invasion of the alien plant *Impatiens glandulifera* (Balsaminaceae) affects pollen transfer at the community level. We asked whether more alien pollen is deposited on the stigmas of plants on invaded sites, whether deposition is affected by stigma type (dry, semidry and wet) and whether the invasion of *I*. *glandulifera* changes the structure of the resulting pollen transfer networks. We sampled stigmas of plants on 10 sites invaded by *I*. *glandulifera* (hereafter, balsam) and 10 non-invaded control sites. All 20 networks had interactions with balsam pollen, although significantly more balsam pollen was found on plants with dry stigmas in invaded areas. Balsam pollen deposition was restricted to a small subset of plant species, which is surprising because pollinators are known to carry high loads of balsam pollen. Balsam invasion did not affect the loading of native pollen, nor did it affect pollen transfer network properties; networks were modular and poorly nested, both of which are likely to be related to the specificity of pollen transfer interactions. Our results indicate that pollination networks become more specialized when moving from the flower visitation to the level of pollen transfer networks. Therefore, caution is needed when inferring pollination from patterns of insect visitation or insect pollen loads as the relationship between these and pollen deposition is not straightforward.

## Introduction

Human activities have significantly increased the introduction and movement of alien species around the planet since the 18^th^ Century [1]. Although alien species are not always detrimental to the local community, indeed they sometimes cause positive [2] or neutral effects [3,4]; some alien species can potentially become invasive and outcompete native species causing substantial changes to ecological processes [1,5,6]. Pollination of native species, for example, can be affected by plant invasion due to reduced visits of pollinator insects [6], which can lead to decreased seed set [7–10], the latter occurring by either reduced transfer of conspecific pollen or excessive heterospecific pollen deposition from the alien plant [11,12]. Although alien pollen deposition on native stigmas is considered one of the main causes of pollination disruption worldwide [5,13] and while there have been recent attempts to understand pollen transfer at the community level [14–17], there are still large gaps in our understanding of the impact of alien plants on pollination. A pioneering study by Fang and Huang (2013) [15] focusing on plant-plant directed networks showed that heterospecific pollen transfer was common and that stigma position is a major floral trait determining the role of a species within the network as pollen receiver or donor. Although a novel and exciting approach which lead to the publication of the first pollen transfer network, this study was from a single field plot and data were collected over just two consecutive days. Here we adapt and expand their approach to investigate effects of a highly invasive plant species, *Impatiens glandulifera*, on the structure of bipartite pollen transfer networks. In contrast to Fang and Huang (2013) [15] where links were established between plant species that share the same pollen type deposition, we constructed bipartite networks where links are established between plant species and the pollen types deposited in its stigmas, analysed at the community level.

Alien plants can integrate into native communities by establishing new interactions with generalist pollinators [6,18]. Sharing pollinators makes it likely that alien pollen will be dispersed throughout the whole community [6,19]. This is even more likely if the alien plant shows a “magnet effect”, characterized by bigger and more colourful flowers, and high amounts of nectar and pollen production [2,10] thereby attracting large numbers of pollinators. Lopezaraiza-Mikel et al. (2007) [19] found 95.5% of pollen on flower visitors was from the alien balsam plants in invaded areas whilst in the adjacent non-invaded areas the pollen network was 35% alien pollen, indicating that pollinators can carry alien pollen into nearby non-invaded areas. What is not known currently is whether this pollen is then deposited on the stigmas of native plants.

The vast majority of data concerning pollination at the community level is based on observations of animal species visiting flower species, which are gathered as plant-flower visitor networks [20–22]. These visits will not necessarily translate into pollination due to, for example, pollen loss and nectar thieves [23]. The key step in pollination happens on the stigma where the pollen grains are deposited and the physical and molecular reactions, which allow pollen grains to germinate, take place. Therefore, measures of pollen transfer to stigmas of plants at the community level are a step closer to the outcome of pollination, i.e. seed set. Both pollen grains and stigmas show great variety of shapes, sizes and structures [17,24–26]. Stigmas vary in secretion level and are classified as wet (those that produce fluid secretions), dry (no secretions produced), and semidry (an intermediate state between wet and dry, recently proposed for the Asteraceae family [27,28]). Flowers with wet stigmas are believed to be more prone to higher heterospecific pollen deposition (due to their richly hydrated surface) than those with dry stigmas [24]. Therefore, we expect that plant species with wet stigmas (which is also the stigma type of balsam) will receive higher amounts of balsam pollen grains.

In what follows we present data from a well replicated field study consisting of ten sites invaded by balsam and ten control sites. Our objectives are twofold: 1) to quantify the amount of balsam, conspecific and heterospecific pollen deposition on stigmas of native plants, and to test whether this is affected by stigma type, and 2) to test whether the presence of a highly invasive alien plant changes the structure of the pollen transfer networks.

## Materials and Methods

### The focal plant: *Impatiens glandulifera* Royle (Balsaminaceae)

*Impatiens glandulifera*, known as Himalayan balsam or balsam, is an alien plant that has spread successfully through Europe, integrating easily into native pollinator communities on account of its highly rewarding flowers [10,19]. Balsam has a rate of sugar production an order of magnitude higher than most UK native plant species (Balsam: 11312 μg in 24h versus *Stachys palustris*: 1384, *Geranium robertianum*: 811, *Silene dioica*: 714, *Trifolium pratense*: 400, *Brassica napus*: 362, *Trifolium repens*: 129, *Epilobium hirsutum*: 40 [29]). Therefore, balsam flowers are visited by an array insect species such as *Bombus* sp. and *Apis mellifera* (Hymenoptera), *Platycheirus albimanus*, *Episyrphus balteatus* and *Melangyna* sp. (Syrphidae), *Halictus tumulorum* (Halictidade), and *Rhinophora lepida* (Rhinophoridae) [19,30] which makes it likely that balsam share pollinators with native plant species. Furthermore, while early work suggested that balsam competes with native plants for pollinators [8], more recent work rather suggests that it increases the number of flower visitors to native plants by facilitation [12,19].

### Study area and sampling design

The study was carried out in Bristol, UK (51°27`N, 2°35`W) in ten areas invaded by balsam, and ten adjacent non-invaded control areas with comparable plant communities (S1 Fig). At the invaded sites balsam was the dominant plant species covering at least 70% of the study area. Non-invaded sites were located at least 500 m from sites with balsam (mean = 1939.7 m [min = 500 m; max = 4963 m]). The vegetation of both invaded and non-invaded study sites comprises a range of habitats, from grasslands, to meadows and woodlands, occurring on city parks, along walking paths, on the edge of rivers, and also in nature reserves. Permissions for sampling were obtained from the Bristol City Council and from the Wildlife Trust. Field work and sampling did not involve endangered or protected species. We sampled each site once, in a single visit from late July to late August 2013, this covering the start of, and the main flowering period, of balsam.

At each site, a point sampling approach was used along a 50 m transect whereby a marker stick was placed every meter along the trail and from each plant found within a 30 cm radius of this point, we collected three open flowers. These were placed in small vials with the stem submerged in water for transport to the lab. If three flowers were not available we collected those present (i.e. one or two) and our response variable is the average pollen load of these stigmas. In the case of composite inflorescences, the three stigmas were collected in a single floral unit, one in the middle, and the other two on opposite external sides of the inflorescence. In the lab, stigmas were carefully removed from the flowers with clean tweezers and mounted on a slide with fuchsin jelly [31]. Finally, all plant species were classified according to the type of stigma: wet, dry or semi-dry [24,27]. To check that our data on pollen transfer were not influenced by variation in plant species richness and abundance among study sites, we tested for differences in plant stigma species richness, pollen type richness and plant abundance (estimated by the number of samples per species) between the invaded and non-invaded sites with a one-way analysis of variance. Given that we were interested in the effect of balsam on native stigmas and not vice versa, we did not collect balsam stigmas.

### Pollen identification

Prior to pollen analyses, we built a pollen reference collection by collecting pollen from the anthers of species occurring within and around our study sites. The pollen grains on each stigma were counted and identified, either to species, genus or family level. If this was not possible, they were morphotyped using size, shape and exine structure as distinguishing characteristics. Although not all pollen grains were easily identified to species, the balsam pollen grains were distinct from all others. The pollen grains on each stigma were classified according to their origin: conspecific (their own species of pollen grains), balsam or heterospecific (pollen grains from all other species except balsam) in order to test for differences in pollen deposition on the different types of stigma.

### Objective 1: to quantify the amount of balsam, conspecific and heterospecific pollen deposition on stigmas of native plants, and to test whether this is affected by stigma type

In order to determine if there were differences in pollen deposition (balsam, conspecific, heterospecific) between habitats and among stigma types we used generalized linear mixed models (GLMMs) with a gamma error distribution (link = log). The use of GLMMs here is expected to solve the problem of pseudo-replication since data collected within sites are not independent, with multiple plant species sampled at each site, all of which experience the same environmental conditions, and face similar levels of resource availability and competition. We chose the gamma error distribution because it deals with right-skewed data that cannot assume values less than zero. A GLMM was fitted for each of the pollen origins (e.g. three different models) applying the glmer function from the lme4 package in R [32]. Habitat (invaded, non-invaded), stigma type (wet, dry, semi-dry) and their interaction were modelled as fixed effects, whilst stigma species and sampling site were modelled as a random effects, to control for variation of species composition and abundance among sites. The significance of terms in the models was assessed using likelihood ratio tests comparing models with and without the fixed effect of interest [33] and Tukey pairwise comparisons made using the glht function with the multcomp package [34]. Predictions from GLMM models were plotted with standard errors based on simulation using the arm package [35]. A variance components analysis was also performed by fitting intercept-only versions of the models, with stigma type and habitat as random factors, alongside site and species [36]; this allowing us to estimate the proportion of the variance in the observed pollen deposition associated with the different factors [36].

### Objective 2: To test whether the presence of a highly invasive alien plant changes the structure of pollen transfer networks

Pollen transfer networks were used to visualize which plant stigma species have which pollen types deposited on their surface. These networks link plant species if the pollen of one species is found on the stigma of another species and they provide community level information on pollen movement. Networks were constructed for each of the 20 study sites, organized as bipartite *m* x *n* quantitative matrices, with *m* plant stigma species and *n* pollen types. The elements in the matrix represented the average number of pollen grains of a given pollen types *n* found on a given stigma species *m*. Changes in pollen transfer interactions and network structure following invasion by balsam were investigated using the following parameters which quantitatively describe the overall network properties, as well as the level of specialization/generalization of species and interactions: (i) plant stigma species and pollen types richness and abundance (where abundance equals the number of individual plants per 50 x 1 m), (ii) number of interactions; (iii) linkage density: estimates the number of interactions per species divided by the total number of species [37]; (v) weighted connectance–linkage density divided by the number of species in the network [38]; (iv) H`2 specialization–derived from Shannon entropy, this index describes the overall network specialization based on how much the observed interactions deviates from that expected based on their total number of interactions, as such that H`2 increases, from 0 to 1, with network specialization [39]; (v) weighted nestedness–estimates the presence of highly generalist species connecting to less specialist species, this index is based on NODF [40]; (vii) modularity–identifies groups of species that interact more strongly within groups than among groups (modules), here we applied the recent developed metric called QuanBiMo [41], which is a quantitative version of the widely used Newman & Girvan`s algorithm [42].

In order to test whether the network structure changed with invasion we ran individual one-way analysis of variance models for each network properties described above. We fitted habitat (invaded and non-invaded) as an independent variable and each parameter as the response variable in separate models; given that the number of plant stigma species and pollen types was different among the 20 sites and this could potentially affect network properties, we also fitted the models using these parameters as covariates to check for potential confounding effects. Parameters were log-transformed to fit normality assumptions when necessary; otherwise non-parametric Kruskal-Wallis models were fitted to the data when normality was not achieved by data-transformation. Model validation to check for homoscedasticity and normality of the residuals was performed following Zuur at al. (2009) [33], and network metrics were calculated using the bipartite package [43] in R [44]. Species-level analyses were not conducted because the number of plant species was not constant across networks nor was community composition, preventing us from testing the impact of balsam on individual plant species.

## Results

**Fig 1:**
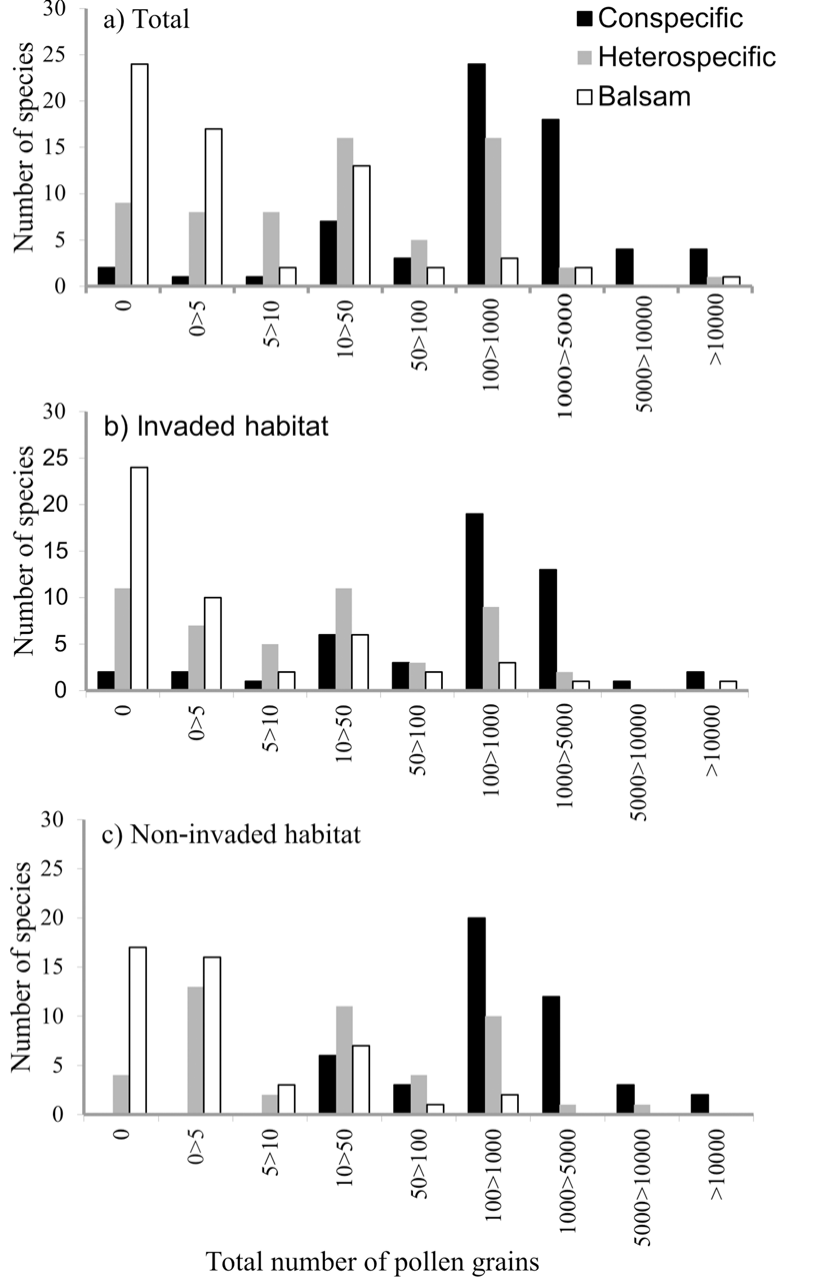
Frequency histograms showing the number of pollen grains per stigma species in (a) both habitats pooled, (b) in invaded habitats and (c) in non-invaded habitats.

Overall we identified 538737 pollen grains on 3855 stigmas. The stigmas were collected from 1469 plants of 64 species. There were 62 pollen types from 21 plant families and a further 27 species of pollen were morphotyped: 18 at genus level, and two at family level. On average, each stigma had a mean of 140 pollen grains on its surface (range 0–6906; median = 12). Most of the pollen grains recorded (83%) were conspecific (total = 448933, mean per stigma = 116.45; range 0–6906; median = 149). A further 58598 pollen grains were heterospecific (mean per stigma = 15; range 0–2451; median = 4) and 31206 balsam pollen grains were recorded (mean per stigma = 8; range 0–2591; median = 8) (S2 Fig). Only two plant species had no conspecific pollen grains on their stigmas, whereas 24 species had no balsam pollen and nine species had no heterospecific pollen (Fig 1 and S3 Fig).

We recorded 34 species with dry stigmas, 19 with semidry, and 11 species with wet stigmas (S1 Table). Aside from balsam, we recorded eight other alien species: *Artemisia vulgaris*, *Brassica napus*, *Buddleja davidii*, *Calystegia sepium*, *Lamium album*, *Malva sylvestris*, *Tripleurospermum inodorum*, and a garden escape *Lantana camara*, all at a much lower density than balsam (S2 Table). The stigmas of plants occurring on invaded sites had significantly higher percentage of balsam pollen deposition compared to non-invaded habitats (invaded: 4.13 ± 13.02; non-invaded = 2.78 ± 9.77 [mean ± SD]); balsam pollen grains were recorded in all sites studied (S2 Fig and S3 Table). In comparison the deposition of conspecific pollen grains (invaded: 79. 68 ± 30.98; non-invaded = 80.82 ± 30.26 [mean ± SD]) and heterospecific pollen grains (invaded: 16.19 ± 27.02; non-invaded = 16.4 ± 27.65 [mean ± SD]) were similar at invaded and non-invaded sites.

### Objective 1: Deposition of pollen grains between habitats and among stigma types

**Fig 2:**
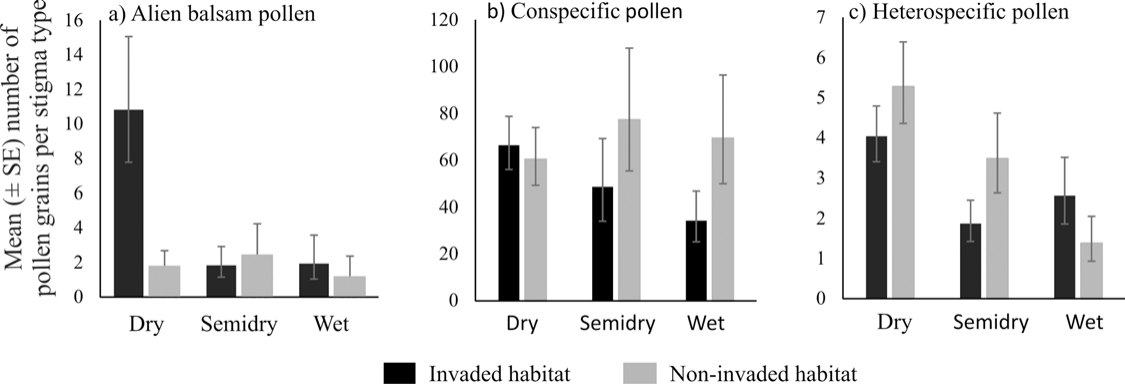
The predicted geometric mean number (± S.E.) of (a) alien balsam (*Impatiens glandulifera*), (b) conspecific and (c) heterospecific pollen grains on dry, wet and semidry stigmas in invaded and non-invaded habitats using generalized linear mixed-effects models. The only significant pairwise difference, based on posteriori tests, was found between dry stigmas in invaded and non-invaded habitats.

A significant interaction was detected between habitat and stigma type for alien balsam pollen deposition (χ^2^ = 6.667 [2], p = 0.036, S4 Table). Post-hoc analyses showed that higher amounts of balsam pollen were deposited on species with dry stigma types in invaded habitats (p = 0.048, Fig 2A and S4 Table). Balsam pollen deposition on wet and semidry stigmas was similar between invaded and non-invaded sites (p > 0.17, Fig 2A and S4 Table). The variance component analysis indicated that the primary source of variation in balsam pollen deposition was plant stigma species (64.43%), compared to 10.58% associated to other factors (habitat and stigma type combined). This result indicates that there is a species-specific effect in place regarding balsam deposition where some species have high amounts of balsam in their stigmas while others very low amounts or none. In fact, we found that only five plant species, from a community of 64 species, had 91% of the total balsam pollen recorded in both habitats on their stigmas. Those species are: *Calystegia sepium* with the highest number of balsam pollen grains per stigma, followed by *Chamerion angustifolium*, *Silene dioica*, *Epilobium hirsutum* and *Circaea lutetiana* (S3 Fig and S5 Table). Of those species, *C*. *angustifolium* and *S*. *dioica* were recorded only in invaded sites, *C*. *lutetiana* was found on five invaded and one non-invaded sites, *C*. *sepium* and *E*. *hirsutum* were recorded in eight invaded sites and five and seven non-invaded sites, respectively (S2 Table).

There was no significant difference in the number of conspecific pollen grains on plant stigmas in invaded and non-invaded habitats or among stigma types (all p > 0.13, Fig 2B, and S4 Table). Plant stigma species explained 65.9% of the variance of conspecific pollen deposition whilst 34.1% remained unexplained at the residual level. Similarly, there was no effect of habitat or stigma type on heterospecific pollen deposition (all p > 0.18, Fig 2C and S4 Table). In this case 56.95% of the variance was due to plant stigma species, 3.48% to stigma type, and 39.56% to the residuals. The species that received the highest level of conspecific pollen deposition (*Vicia sepium*, *Hypericum tetrapterum*, *Epilobium montanum*, *Hypochaeris radicata*, *Buddleja davidii*) were different from those with the highest balsam deposition (*Calystegia sepium*, *Chamerion angustifolium*, *Silene dioica*, *Epilobium hirsutum*, *Circaea lutetiana)*. Two of the species that received highest amount of heterospecific pollen overlapped with the species that received highest amount of balsam pollen (*Calystegia sepium and Silene dioica*) (S5 Table).

### Objective 2: Effects of balsam invasion on the structure of the pollen transfer networks

**Fig 3:**
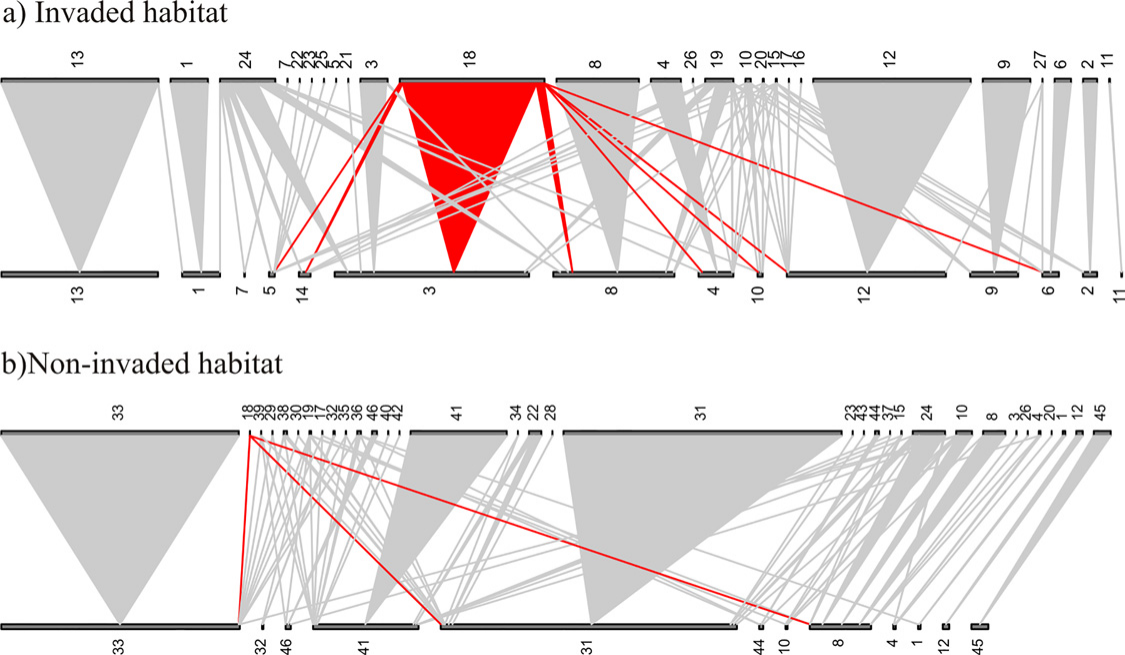
One of the pairs of pollen transfer networks from invaded (top) and non-invaded habitats (bottom). Plant stigma species are shown at the bottom of each network, whilst pollen types are at the top. Plant stigma species and pollen types are represented by rectangles, the size of which reflects their abundance; triangles connect the two if the pollen type is found on the stigma species, and the width of the triangle is related to the frequency of the interaction. Red interactions are those with balsam pollen, i.e. balsam pollen grains were found on the stigmas of the species with which it is linked. Codes for species are: 1- *Arctium minus*; 2 –*Buddleja davidii*; 3- *Calystegia sepium*; 4- *Circaea lutetiana*; 5 –*Cirsium arvense*; 6 –*Clematis vitalba*; 7 –*Dipsacus pilosus*; 8 –*Epilobium hirsutum*; 9 –*Filipendula ulmaria*; 10 –*Heracleum sphondylium*; 11 –*Rosa pimpinellifolia*; 12 –*Rubus fruticosus*; 13 –*Vicia sepium*; 14 –*Silene dioica*; 15 –Asteraceae 1; 16 –*Dipsacus* sp.; 17 –*Epilobium* sp.; 18 –*Impatiens glandulifera*; 19 –Morphotype (hereafter, M) 1; 20 –M10; 21 –M16; 22 –M4; 23 –M5; 24 –M6; 25 –M9; 26 –*Sonchus asper*; 27 *Taraxacum* sp.; 28 –Asteraceae 4; 29 –*Centaurea nigra*; 30 –*Cirsium* sp.; 31 –*Epilobium parviflorum*; 32 –*Geranium robertianum*; 33 –*Hypericum tetrapterum*; 34 –M11; 35 –M12; 36 –M13; 37 –M14; 38 –non identified; 39 –*Silene dioica*; 40 –*Sonchus* sp.; 41 –*Stachys sylvatica*; 42 –*Taraxacum officinale*; 43 –*Trifolium* sp.; 44 –*Trifolium dubium*; 45 –*Trifolium pratense*; 46 –*Trifolium repens*.

There were no significant differences between invaded and non-invaded habitats in any of the network properties analysed (Table 1 and S6 Table; Fig 3 and S4 Fig). In invaded areas, networks contained 12.1 ± 4.38 (mean ± SD) plant stigma species and 24.9 ± 7.94 pollen types, while in non-invaded habitats there were on average 11.0 ± 4.71 plant stigma species and 28.1 ± 9.43 pollen types. There was no significant difference between habitats in plant stigma species and pollen types richness (F_1.18_ = 0.29, p = 0.6; F_1.18_ = 0.13, p = 0.7, respectively), rather habitats differed in the presence of balsam, as was intended by our sampling design. Similarly, the number of individual plants sampled in each site was not significantly different between habitats (invaded = 71.1 ± 19.58; non-invaded = 74.4 ± 21.45; F_1.18_ = 0.13, p = 0.7).

**Table 1:** Descriptive means (± SD) of each network metric in each habitat and the results of the Analysis of Variance testing the effects of the presence of balsam, *Impatiens glandulifera* Royle, on the structure of pollen transfer networks.

	Mean ± SD		Analyses of Variance	
	Invaded	Non-invaded	F_(1,18)_	p-value
Number of interactions	11135.71 ± 5638.23	11148.71 ± 6374.05	2.11e-05	0.99
Linkage density	1.57 ± 0.52	1.43 ± 0.31	0.45	0.51
Connectance	0.05 ± 0.03	0.04 ± 0.02	0.39	0.54
H`2	0.89 ± 0.15	0.96 ± 0.03	1.05^§^	0.30
Nestedness	18.9 ± 9.9	17.05 ±7.45	0.27	0.61
Modularity	0.59 ± 0.16	0.63 ± 0.16	0.5	0.49

^§^ Results are from the Kruskal-Wallis test (χ^2^ and p-value) fitted for the analyses of the non-normal H`2 data.

Pollen transfer networks were on average highly specialized, poorly nested and fairly modular, and communities showed low linkage density and low connectance in both habitats (Table 1). Together these suggest that the overall community tends to specialization in terms of pollen deposition on stigmas. The lack of differences in network structure is explained, at least in part, by the fact that there was no difference in conspecific and heterospecific pollen deposition between habitats and by the fact that balsam pollen deposition was low and species-specific.

## Discussion

Our study uses ecological networks to understand the effect of invasive alien species on pollen transfer interactions at the community level. We found significantly more balsam pollen on plant stigmas in invaded areas; however most of this (91%) was on a very restricted subset of species. We did not find a decrease in conspecific pollen deposition on stigmas in invaded areas. Indeed, we saw only a partial effect of stigma type, and the presence of the alien balsam pollen did not affect the structure of the pollen transfer networks. There is a remarkably low deposition of balsam pollen on most stigmas given the high quantity of balsam pollen transported by flower-visiting insects [19], a finding that is both interesting and counter intuitive. In this section we discuss the limitations of our study and the main results in the light of our two objectives.

### Limitations

There are three main limitations with our approach. First, pollen identification at species level can be challenging when sampled from a community containing many closely related species. However, the invasive species *Impatiens glandulifera* can easily be distinguished from all other species in the community, so we are confident about identification and quantification of its pollen. Some of the native pollen could not be ascribed to a given species though and was identified at the genus or family level (18/64 and 6/64 respectively) or morphotyped (25/64 species). Second, conspecific pollen grains will be from two sources: a) from the sampled plant because flowers were not emasculated, and b) from other plants via pollinators, and these different sources cannot be differentiated. Emasculation was not used as it could have affected insect visitation. Finally, our most interesting comparison is between the data presented here from 2013 and the data from Lopezaraiza-Mikel et al. (2007) [19], although the low balsam pollen deposition in 2013 cannot be compared directly to the high balsam pollen transport seen in 2007 because both studies were run independently. However, we expect that the core species in the networks of both studies remain constant over time, i.e. although some turnover in the rare, less abundant and more specialized species is expected across years, it is very likely that the most common species will be present in both studies (see [45,46]). Furthermore, balsam produces nectar at a rate an order of magnitude higher than native plants along with large quantities of pollen [29] and it is visited by large numbers of insects [19,30,47] which makes it likely that the most abundant and generalist species visiting balsam in 2007 would also be visiting it in 2013.

### Deposition of balsam, conspecific and heterospecific pollen grains between habitats and among stigma types

We recorded significantly more alien balsam pollen grains on plant stigmas in invaded than in non-invaded areas, although deposition of conspecific and heterospecific pollen grains was similar between habitats. Stigma type was important for balsam pollen deposition with most of the pollen being deposited on dry stigmas, even though wet stigmas were expected to receive more balsam pollen due to its liquid-rich surface that is believed to facilitate pollen adhesion and because balsam flowers themselves have wet stigmas [24,25]. Furthermore, balsam pollen deposition was mostly explained by plant stigma species, as shown by the variance component analysis. In the following, we discuss possible explanations for the patterns reported here.

First, sympatric species are assumed to have evolved mechanisms to avoid each other’s heterospecific pollen deposition and to select for conspecific pollen [17]. Balsam is an alien species that did not evolve with the other plant species in the invaded communities, thus does not share the same evolutionary history. Consequently we would not expect native species to have a mechanism to avoid alien balsam pollen deposition. However we found that invasive alien balsam pollen deposition was low or absent for most of the plant species in the community, and that most alien pollen deposition occurred on just a few plant species, suggesting that the native plants at our field sites have a mechanism to avoid alien pollen deposition that may be similar with the mechanism in place to avoid sympatric heterospecific pollen deposition, even under high input from pollinators.

Another aspect is that flower morphology may play an important role in limiting pollen loading on stigmas. According to Montgomery and Rathcke (2012) [14], a restrictive stigma position (those protected by petals and sepals) seems to work as barrier to alien and heterospecific pollen loading, while unrestrictive stigmas (those protruding from the flower) receive more alien and heterospecific pollen grains, and similar amounts of conspecific pollen grains. In our study, the species that received most balsam pollen (i.e. *Calystegia sepium*, *Epilobium hirsutum*, *Circaea lutetiana*, *Chamerion angustifolium*, *Cirsium palustre*) all have protuberant unrestrictive stigmas, although other species, such as *Rubus fruticosus*, *Heracleum sphondylium*, and many of the Asteraceae species (e.g. *Sonchus asper*, *Bellis perennis*, *Centaurea nigra*), which also have unrestrictive stigmas, had low amounts of alien balsam pollen on their stigmas.

Most of the balsam pollen deposition was found on species with dry stigmas and species identity was significant in determining balsam pollen deposition. The species that received the largest quantities of balsam pollen had both wet stigmas (*Circaea lutetiana*, *Chamerion angustifolium* and *Trifolium repens*) and dry stigmas (*Calystegia sepium*, *Epilobium hirsutum* and *Silene dioica*). The results of the mixed models indicated that, although part of the variance was due to stigma type, a high proportion of the variance is not explained by any of the variables we considered. What happens between an insect visit to a balsam flower and the pollen deposition on stigmas is unknown which makes it a challenge to explain the disparity between the high amount of balsam pollen loads on pollinators’ bodies recorded by Lopezaraiza-Mikel et al. (2007) [19] and the low quantity of balsam deposition on plant stigmas. Possible mechanisms underlying these data include pollen loss, stigma/pollen compatibility, and pollinator behaviour. For instance, it is likely that inter and intraspecific variation in insect’ behaviour, such as floral constancy [48,49] and grooming [50], affect the amount of balsam pollen grains loading on insects’ bodies, and consequently influence the quantity of balsam transfer to the stigmas. The mechanism underlying the selectivity of balsam pollen deposition on a subset of stigma species requires further investigation—it may be that other and less well understood factors are influencing balsam pollen deposition, for example the recent discovery of the importance of electrostatic forces in plant-pollinator interactions [51]. Although logistically challenging, a possible solution here would be to sample pollen visitation, pollen transport and pollen transfer networks concomitantly, recording the interactions and pollinators behaviour as they switch from flower to flower.

### Effects of balsam invasion on the structure of pollen transfer networks

While all twenty networks contained balsam pollen, balsam was integrated in the community via considerable pollen deposition on stigmas of a few plant species and rare deposition on stigmas of the remainder. The similar amount of conspecific and heterospecific pollen deposition in the invaded and non-invaded habitats explains the lack of significant differences between habitats in the network metrics analysed. Linkage density and interaction evenness, which describe the distribution of links in the networks, were statistically similar between habitats suggesting that, quantitatively, the way species interact in terms of pollen transfer has not been affected by balsam invasion. Similarly, because the interactions between pollen and stigma species remained fairly constant between habitats (because balsam interactions were restricted to a small subset of species), the structure of the network described by modularity, nestedness and specialization H`2 was robust to invasion.

Contrary to the most plant-flower visitor networks in which nestedness is a common pattern that depicts the presence of generalist species that interact with other generalist species as well as with a subset of more specialist ones, our pollen transfer networks showed rather low values of nestedness. This has two main implications: first, from the plants point of view, generalist plant species that are expected to be visited by an array of generalist pollinator species could be expected to donate and receive large amounts of heterospecific pollen grains. This was not the case though in our specialized and modular pollen transfer networks in which conspecific pollen grains were the main interactions recorded in all stigma species. Second, the high values of specialization depicted by the specialization index H`2 and the modularity algorithm point toward a highly specialized system which contradicts the current knowledge on pollination networks been composed of highly generalist species connecting sub-groups of more specialist ones. Our findings have important implications for how pollination networks are sampled and indicate that the pollination is more specialized than visitation data suggests. Ballantyne et al. (2015) [52] reached the same conclusion when studying single visit deposition of pollen grains as the interaction links. These new approaches that look at pollen transfer rather than just visitation or pollen transport are likely to provide new insights when asking whether invasive species are a threat to pollination systems.

Similar to our findings, Fang and Huang’s (2013) [15] plant-plant network also reported that while heterospecific pollen transfer was common, it was at low abundance. They also found that most plant species were relatively specialized in pollen transfer, and even those plants considered generalist in terms of pollinators’ visits did not necessarily donate pollen to other species. Thus given that alien and heterospecific pollen grains deposition on stigmas seems to occur at very low rates in natural communities, it seems unlikely that the stigma clogging of native plant species previously considered to be one of the main mechanisms driving the negative effects of alien plants on native communities [26,53] is a widespread problem.

Plant-visitor networks are in general robust to the loss of species [54,55]. In contrast, little is known about how robust networks are to the gain of species and how native species rewire to incorporate novel interactions. The effects of adding alien species on plant-pollinator networks was modelled recently using the balsam visitation data of Lopezaraiza-Mikel al. (2007) [19] and demonstrated that the effects of introduced species on network structure depends on the number and frequency of the interactions that the novel species is able to establish in the community, and whether the novel species compete or add new interactions [56]. Here we have shown that the effects of an alien plant invasion do not affect the overall interactions of pollen transfer at the community level, rather its effects impact on just a handful of species.

### Final remarks and further steps

Although the structure of the pollen transfer networks did not change with the invasion of the alien plant *Impatiens glandulifera*, it is still too early to say that alien species do not have an impact on pollination as further studies are needed to better understand general patterns at the stigma level regarding alien pollen inputs and the robustness of pollen transfer networks to the invasion of alien species. Furthermore, balsam pollen deposition on plant stigmas seems to be species-specific, indicating that there are effects on some plant species. Given the readiness of pollination community ecologists to consider visitation as a surrogate measure of pollination, the results presented here, along with those of Fang and Huang (2013) [15], strongly suggest that greater caution needs to be taken before assuming that this is the case. What happens on the stigma surface is an important step in understanding the impact of alien plants as this is where pollen discrimination occurs, and where pollination in terms of seed production ultimately takes place. To progress this field of research, more collaboration is needed between plant scientists who work on the molecular and physiological mechanisms of pollen transfer interactions and the field ecologists who work on pollination and alien plants. We predict that such new collaborations will shed considerable insight into the subtle complexities of plant-pollinator interactions.

## Supporting Information

S1 Table Types of stigma following Heslop-Harrison & Shivanna (1977), and Hiscock et al. (2002). (DOCX)

S2 Table List of plant species in which stigmas where collected from found in each of the 20 study sites. Asterisks (*) indicate alien species, according to the “GB non-native species secretariat”, available online at http://www.nonnativespecies.org/home/index.cfm.
(DOCX)

S3 Table Mean number of balsam (*Impatiens glandulifera*) pollen grains found per species in each site in each habitat. Empty cells mean that the stigma species was not recorded in that respective site. Values of zero mean that the stigma species was recorded in the respective site but had no balsam pollen deposition on the stigmas.
(DOCX)

S4 Table Results of the Generalized Linear Mixed Models (GLMM`s) and Post-hoc tests (Tukey) testing whether the deposition of balsam (*Impatiens glandulifera*), conspecific and heterospecific pollen grains on stigmas are different between invaded and non-invaded sites, and whether it is affected by the stigma type (dry, semidry, wet). Full model: y = meanpollen ~ habitat*stigmatype + (1|sitecode/stigmaspecies), family = Gamma (link = log)
(DOCX)

S5 Table The five species with the highest mean deposition of pollen grains per stigma, according to each type of pollen: conspecific, balsam (*Impatiens glandulifera*) and heterospecific. The mean is the total number of pollen grains counted on the stigmas of each species divided by the total number of stigmas sampled, with data pooled from the 20 sites.
(DOCX)

S6 Table Results of the Analysis of Variance testing the effects of the presence of balsam, *Impatiens glandulifera*, on the structure of pollen transfer networks. Values in bold are significant results at p < 0.05. The results show the significant p values for each term in the full model which has habitat (p_habitat_) as the main effect and stigma (p_stigmasp_) and pollen species richness (p_pollensp_) as covariates. ^§^ Results of a Kruskal-Wallis test fitted to the non-normal H`2 data; each p-value correspondent to an independent non-parametric Kruskal-Wallis test.
(DOCX)

S1 Fig Map of the study area around the city of Bristol, UK. Red points are areas invaded by balsam (*Impatiens glandulifera*); blue areas are areas non-invaded by balsam, and used as “control” plots. Figure created using OpenStreetMap for illustrative purposes only. Data is available under the Open Database License.
(DOCX)

S2 Fig Distribution of balsam (*Impatiens glandulifera*) pollen grains found on stigmas of plants occurring in invaded and non-invaded habitats. Upper panels show the distribution of frequencies of the number of balsam pollen grains per sample in each habitat. Lower panels show the distribution of balsam pollen grains per sample across the different sites in each habitat.
(DOCX)

S3 Fig Frequency histograms showing the number of pollen grains per stigma species; the pollen grains are classified as (a) conspecific, (b) balsam (*Impatiens glandulifera*) and (c) heterospecific (pollen grains of all other species found in the 20 study sites). (DOCX)

S4 Fig Pollen transfer networks invaded and non-invaded by balsam, *Impatiens glandulifera* Royle. Networks are shown in pairs, in the order the data was collected. Top species are pollen grains; bottom species are stigma species. The width of the rectangles in top and bottom side of the network and the width of the triangles linking both sides represent the abundance of each species and the frequency of interactions, respectively. Red interactions are those with *I*. *glandulifer*a.
(DOCX)

S1 Dataset Dataset used to build pollen transfer networks presented at the research article: Emer, C, IP Vaughan, S Hiscock and J Memmott (2015). The impact of the invasive alien plant, *Impatiens glandulifera*, on pollen transfer networks. PlosOne 00:0000.
(TXT)
