# Supplementary material for: The Impact of the Invasive Alien Plant, Impatiens glandulifera, on Pollen Transfer Networks
Source: PLoS One. 2015 Dec 3;10(12):e0143532. doi: 10.1371/journal.pone.0143532 (PMC4669169; doi:10.1371/journal.pone.0143532)
Supplement: S1 Table — (DOCX) [file pone.0143532.s001.docx]

**S1 Table. Types of stigma following Heslop-Harrison & Shivanna (1977), and Hiscock et al. (2002).**

|  | **Stigma type** | | |
| --- | --- | --- | --- |
|  | **Dry** | **Semidry** | **Wet** |
| *Achillea millefolium* |  | x |  |
| *Alliaria petiolata* | x |  |  |
| *Angelica sylvestris* |  |  | x |
| *Anthriscus sylvestris* | x |  |  |
| *Arctium minus* |  | x |  |
| *Artemisia vulgaris* |  | x |  |
| *Bellis perennis* |  | x |  |
| *Brassica sp* | x |  |  |
| *Brassica napus* | x |  |  |
| *Brassica* sp1 | x |  |  |
| *Brassica* sp2 | x |  |  |
| *Buddleja davidii* |  |  | x |
| *Calystegia sepium* | x |  |  |
| *Capsella bursa-pastoris* | x |  |  |
| *Centaurea nigra* | x |  |  |
| *Chamerion angustifolium* |  | x |  |
| *Circaea lutetiana* |  |  | x |
| *Cirsium arvense* |  | x |  |
| *Cirsium palustre* |  | x |  |
| *Cirsium vulgare* |  | x |  |
| *Clematis vitalba* | x |  |  |
| *Convolvulus arvensis* | x |  |  |
| *Dipsacus fullonum* | x |  |  |
| *Dipsacus pilosus* | x |  |  |
| *Epilobium hirsutum* | x |  |  |
| *Epilobium montanum* | x |  |  |
| *Epilobium parviflorum* | x |  |  |
| *Eupatorium cannabium* | x |  |  |
| *Filipendula ulmaria* | x |  |  |
| *Galium aparine* | x |  |  |
| *Geranium columbinum* | x |  |  |
| *Geranium robertianum* | x |  |  |
| *Geum urbanum* | x |  |  |
| *Heracleum sphondylium* |  |  | x |
| *Hypericum tetrapterum* | x |  |  |
| *Hypochaeris radicata* |  | x |  |
| *Impatiens glandulifera* |  |  | x |
| *Lamium album* | x |  |  |
| *Lantana camara* |  |  | x |
| *Lapsana communis* |  | x |  |
| *Malva sylvestris* | x |  |  |
| *Matricaria matricarioides* |  | x |  |
| *Myosoton aquaticum* | x |  |  |
| *Polygonum aviculare* | x |  |  |
| *Prunella vulgaris* | x |  |  |
| *Pulicaria dysenterica* |  | x |  |
| *Ranunculus repens* | x |  |  |
| *Rosa pimpinellifolia* | x |  |  |
| *Rubus fruticosus* | x |  |  |
| *Rubus sp1* | x |  |  |
| *Senecio erucifolius* |  | x |  |
| *Senecio jacobaea* |  | x |  |
| *Silene dioica* | x |  |  |
| *Solanum dulcamara* |  |  | x |
| *Sonchus asper* |  | x |  |
| *Sonchus oleraceus* |  | x |  |
| *Stachys palustris* | x |  |  |
| *Stachys sylvatica* | x |  |  |
| *Stellaria media* | x |  |  |
| *Taraxacum officinale* |  | x |  |
| *Trifolium dubium* |  |  | x |
| *Trifolium pratense* |  |  | x |
| *Trifolium repens* |  |  | x |
| *Tripleurospermum inodorum* | | x |  |
| *Vicia sepium* |  |  | x |
