# Supplementary material for: The Impact of the Invasive Alien Plant, Impatiens glandulifera, on Pollen Transfer Networks
Source: PLoS One. 2015 Dec 3;10(12):e0143532. doi: 10.1371/journal.pone.0143532 (PMC4669169; doi:10.1371/journal.pone.0143532)
Supplement: S2 Table — Asterisks (*) indicate alien species, according to the “GB non-native species secretariat”, available online at http://www.nonnativespecies.org/home/index.cfm. (DOCX) [file pone.0143532.s002.docx]

**S2 Table. List of plant species in which stigmas were collected from found in each of the 20 study sites**. Asterisks (*) indicate alien species, according to the “GB non-native species secretariat”, available online at http://www.nonnativespecies.org/home/index.cfm.

|  | **INVADED** | | | | | | | | | | **NON-INVADED** | | | | | | | | | |  |
| --- | --- | --- | --- | --- | --- | --- | --- | --- | --- | --- | --- | --- | --- | --- | --- | --- | --- | --- | --- | --- | --- |
| **Stigma species/Site** | **1a** | **2a** | **3a** | **4a** | **5a** | **6a** | **7a** | **8a** | **9a** | **10a** | **1b** | **2b** | **3b** | **4b** | **5b** | **6b** | **7b** | **8b** | **9b** | **10b** | **Total of sites** |
| *Achillea millefolium* |  |  |  |  |  |  | X |  |  |  |  | X |  |  |  | X |  |  |  |  | 3 |
| *Alliaria petiolata* |  |  |  |  |  |  |  |  |  |  |  |  |  |  |  |  |  |  |  | X | 1 |
| *Angelica sylvestris* |  |  |  |  |  |  |  |  | X |  |  |  |  |  |  |  |  |  |  |  | 1 |
| *Anthriscus sylvestris* |  |  |  |  |  | X |  |  |  |  |  |  |  |  |  |  |  |  |  |  | 1 |
| *Arctium minus* |  |  | X | X | X | X |  |  |  |  |  |  | X |  |  |  |  |  |  |  | 5 |
| *Artemisia vulgaris** |  |  |  |  |  |  | X |  |  |  |  |  |  |  |  |  |  |  |  |  | 1 |
| *Bellis perennis* |  |  |  |  |  |  | X | X |  |  |  |  |  |  |  | X | X | X |  |  | 5 |
| *Brassica* sp |  |  |  |  |  |  |  |  |  |  |  |  |  |  |  | X |  |  |  |  | 1 |
| *Brassica napus** |  |  |  |  |  | X |  |  |  |  |  |  |  |  |  |  |  |  |  |  | 1 |
| *Brassica* sp1 |  | X |  |  |  |  |  |  |  |  |  |  |  |  |  |  |  |  |  |  | 1 |
| *Brassica* sp2 |  |  |  |  |  |  |  |  |  |  |  |  |  |  |  |  |  |  |  | X | 1 |
| *Buddleja davidii** |  |  | X |  |  |  | X |  |  |  |  | X |  |  |  | X |  |  |  |  | 4 |
| *Calystegia sepium* * | X | X | X |  | X | X | X | X | X |  | X |  |  |  |  | X | X | X | X |  | 13 |
| *Capsella bursa-pastoris* |  |  |  |  | X | X |  | X |  |  |  |  |  |  |  | X |  |  |  |  | 4 |
| *Centaurea nigra* |  |  |  |  | X |  |  |  |  |  |  | X |  |  | X |  |  |  |  |  | 3 |
| *Chamerion angustifolium* |  |  |  | X | X |  |  |  |  |  |  |  |  |  |  |  |  |  |  |  | 2 |
| *Circaea lutetiana* | X |  | X |  |  |  | X | X | X | X |  |  | X |  |  |  |  |  |  |  | 7 |
| *Cirsium arvense* |  | X | X |  |  | X | X |  | X |  | X | X |  |  | X |  | X | X | X |  | 11 |
| *Cirsium palustre* |  |  |  | X |  |  |  |  |  |  |  |  |  | X |  |  |  |  |  |  | 2 |
| *Cirsium vulgare* |  |  |  |  |  |  | X |  |  |  | X |  |  |  |  | X |  |  |  |  | 3 |
| *Clematis vitalba* |  |  | X |  |  | X |  |  |  |  |  | X |  |  |  | X | X | X |  |  | 6 |
| *Convolvulus arvensis* |  |  |  |  |  |  |  |  |  |  |  |  |  |  |  |  |  |  | X |  | 1 |
| *Dipsacus fullonum* |  |  |  |  |  |  |  |  |  |  |  | X |  |  |  |  |  |  |  |  | 1 |
| *Dipsacus pilosus* |  |  | X |  |  |  |  |  |  |  |  |  |  |  |  |  |  |  |  |  |  |
| *Epilobium hirsutum* | X | X | X |  | X | X | X | X | X |  | X | X | X |  | X |  | X | X | X |  | 15 |
| *Epilobium montanum* | X |  |  |  |  |  |  |  | X |  |  |  |  |  |  |  |  |  |  |  | 2 |
| *Epilobium parviflorum* |  |  |  | X |  |  |  |  |  |  |  |  | X |  |  |  |  |  |  |  | 2 |
| *Eupatorium cannabium* |  |  |  |  |  |  |  | X |  |  |  | X |  |  |  |  |  |  |  |  | 2 |
| *Filipendula ulmaria* |  |  | X |  |  |  |  |  |  |  |  |  |  |  |  |  |  |  |  |  | 1 |
| *Galium aparine* | X |  |  |  | X |  |  |  |  |  |  |  |  |  |  |  |  | X |  |  | 3 |
| *Geranium columbinum* |  |  |  |  |  |  |  |  |  |  |  |  |  |  |  | X |  |  |  |  | 1 |
| *Geranium robertianum* | X |  |  |  |  |  |  | X | X |  |  |  | X |  |  |  |  |  |  |  | 4 |
| *Geum urbanum* | X | X |  |  |  |  |  | X | X | X |  |  |  |  |  |  |  | X |  | X | 7 |
| *Heracleum sphondylium* |  | X | X | X | X | X |  | X | X | X | X |  | X | X |  |  | X |  |  |  | 12 |
| *Hypericum tetrapterum* |  |  |  |  |  |  |  |  |  |  |  |  | X |  |  |  |  |  |  |  | 1 |
| *Hypochaeris radicata* |  |  |  |  | X | X | X |  |  |  |  | X |  |  |  | X | X |  | X |  | 7 |
| *Lamium album** |  |  |  |  |  |  |  | X |  |  |  |  |  |  |  | X |  | X |  |  | 3 |
| *Lantana camara* * | X |  |  |  |  |  |  |  |  |  |  |  |  |  |  |  |  |  |  |  | 1 |
| *Lapsana communis* |  |  |  |  |  |  |  |  |  | X |  |  |  |  |  |  |  |  |  | X | 2 |
| *Malva sylvestris** |  |  |  |  |  |  |  |  |  |  |  |  |  |  |  |  |  |  |  | X | 1 |
| *Matricaria matricarioides* |  |  |  | X | X | X |  | X | X |  |  |  |  |  |  |  |  |  |  |  | 5 |
| *Myosoton aquaticum* |  |  |  |  |  | X |  |  |  |  |  |  |  |  |  |  |  |  |  |  | 1 |
| *Polygonum aviculare* |  |  |  | X |  |  |  |  |  |  |  |  |  |  |  |  |  |  |  |  | 1 |
| *Prunella vulgaris* |  |  |  |  |  |  | X |  |  |  |  |  |  |  |  |  | X | X |  |  | 3 |
| *Pulicaria dysenterica* |  |  |  |  |  |  |  |  |  |  |  |  |  |  | X |  |  |  |  |  | 1 |
| *Ranunculus repens* |  |  |  | X | X |  | X | X |  |  | X |  |  | X |  | X | X | X |  |  | 9 |
| *Rosa pimpinellifolia* |  |  | X |  |  |  |  |  |  |  |  |  |  |  |  |  |  |  |  |  | 1 |
| *Rubus fruticosus* | X |  | X | X | X | X | X | X | X |  | X | X | X |  | X | X | X | X | X |  | 16 |
| *Rubus* sp1 |  |  |  |  |  | X | X |  |  |  |  |  |  |  |  |  | X | X |  |  | 4 |
| *Senecio erucifolius* |  |  |  |  |  |  |  |  |  |  |  |  |  |  |  | X |  |  |  |  | 1 |
| *Senecio jacobaea* | X |  |  | X | X |  | X |  |  |  |  | X |  |  | X |  |  |  |  |  | 6 |
| *Silene dioica* |  |  | X |  |  |  |  |  |  |  |  |  |  |  |  |  |  |  |  |  | 1 |
| *Solanum dulcamara* |  |  |  |  |  |  |  |  |  |  |  |  |  |  |  |  |  | X |  |  | 1 |
| *Sonchus asper* |  |  |  |  |  |  |  |  |  |  |  |  |  |  |  | X |  |  |  |  | 1 |
| *Sonchus oleraceus* |  |  |  |  |  |  |  |  |  |  |  |  |  |  |  |  |  |  |  | X | 1 |
| *Stachys palustris* |  |  |  |  |  |  |  |  |  |  | X |  |  |  |  |  |  |  |  |  | 1 |
| *Stachys sylvatica* |  |  |  |  |  |  | X | X | X |  |  |  | X |  |  | X | X | X |  |  | 7 |
| *Stellaria media* |  |  |  |  |  |  |  |  | X |  |  |  |  |  |  |  |  |  |  |  | 1 |
| *Taraxacum officinale* |  |  |  |  | X | X |  |  |  |  |  |  |  |  |  | X | X | X |  | X | 6 |
| *Trifolium dubium* | X |  |  |  |  |  |  |  |  |  |  |  | X |  |  |  |  |  |  |  | 2 |
| *Trifolium pratense* |  |  |  |  |  | X | X | X |  |  | X | X | X | X | X | X | X | X |  |  | 11 |
| *Trifolium repens* |  |  |  |  |  |  |  |  |  |  |  | X | X |  | X |  | X | X | X |  | 6 |
| *Tripleurospermum inodorum** |  |  |  |  |  | X |  |  |  |  |  |  |  |  |  |  |  |  |  |  | 1 |
| *Vicia sepium* |  |  | X |  |  |  |  |  |  |  |  |  |  |  |  |  |  |  |  |  | 1 |
