# Supplementary material for: The Impact of the Invasive Alien Plant, Impatiens glandulifera, on Pollen Transfer Networks
Source: PLoS One. 2015 Dec 3;10(12):e0143532. doi: 10.1371/journal.pone.0143532 (PMC4669169; doi:10.1371/journal.pone.0143532)
Supplement: S3 Table — Empty cells mean that the stigma species was not recorded in that respective site. Values of zero mean that the stigma species was recorded in the respective site but had no balsam pollen deposition on the stigmas. (DOCX) [file pone.0143532.s003.docx]

**S3 Table. Mean number of balsam (*Impatiens glandulifera*) pollen grains found per species in each site in invaded and non-invaded habitat**. Empty cells mean that the stigma species was not recorded in that respective site. Values of zero mean that the stigma species was recorded in the respective site but had no balsam pollen deposition on the stigmas.

**Table S3.Part 1/2**

|  | **Invaded sites** | | | | | | | | | | **Total on invaded plots** |
| --- | --- | --- | --- | --- | --- | --- | --- | --- | --- | --- | --- |
| **Stigma species/ Site code** | **1** | **4** | **5** | **8** | **10** | **11** | **14** | **16** | **18** | **19** |  |
| *Achillea millefolium* |  |  |  |  |  |  | 0.0 |  |  |  | 0.0 |
| *Alliaria petiolata* |  |  |  |  |  |  |  |  |  |  |  |
| *Angelica sylvestris* |  |  |  |  |  |  |  |  | 0.0 |  | 0.0 |
| *Anthriscus sylvestris* |  |  |  |  |  | 0.0 |  |  |  |  | 0.0 |
| *Arctium minus* |  |  | 0.0 | 34.0 | 1.7 | 47.3 |  |  |  |  | 83.0 |
| *Artemisia vulgaris* |  |  |  |  |  |  | 0.0 |  |  |  | 0.0 |
| *Bellis perennis* |  |  |  |  |  |  | 0.0 | 0.0 |  |  | 0.0 |
| *Brassica* sp |  |  |  |  |  |  |  |  |  |  |  |
| *Brassica napus* |  |  |  |  |  | 25.7 |  |  |  |  | 25.7 |
| *Brassica* sp1 |  | 0.0 |  |  |  |  |  |  |  |  | 0.0 |
| *Brassica* sp2 |  |  |  |  |  |  |  |  |  |  |  |
| *Buddleja davidii* |  |  | 0.0 |  |  |  | 0.0 |  |  |  | 0.0 |
| *Calystegia sepium* | 10328.0 | 3401.0 | 1968 |  | 28.0 | 457.0 | 134.0 | 24.0 | 52.0 |  | 16932 |
| *Capsella bursa-pastoris* |  |  |  |  | 0.0 | 0.0 |  | 0.0 |  |  | 0.0 |
| *Centaurea nigra* |  |  |  |  | 88.8 |  |  |  |  |  | 88.8 |
| *Chamerion angustifolium* |  |  |  | 1276 | 7 |  |  |  |  |  | 1283 |
| *Circaea lutetiana* | 923.0 |  | 0.7 |  |  |  | 0.0 | 0.0 | 0.7 | 1.7 | 926.0 |
| *Cirsium arvense* |  | 22.0 | 1.7 |  |  | 2.3 | 0.7 |  | 2.0 |  | 28.7 |
| *Cirsium palustre* |  |  |  | 390.3 |  |  |  |  |  |  | 390.3 |
| *Cirsium vulgare* |  |  |  |  |  |  | 9.3 |  |  |  | 9.3 |
| *Clematis vitalba* |  |  | 0.3 |  |  | 0.0 |  |  |  |  | 0.3 |
| *Convolvulus arvensis* |  |  |  |  |  |  |  |  |  |  |  |
| *Dipsacus fullonum* |  |  |  |  |  |  |  |  |  |  |  |
| *Dipsacus pilosus* |  |  | 0.0 |  |  |  |  |  |  |  | 0.0 |
| *Epilobium hirsutum* | 271.0 | 825.5 | 109.2 |  | 59.3 | 215.3 | 534.0 | 204.5 | 731.0 |  | 2949.8 |
| *Epilobium montanum* | 0.0 |  |  |  |  |  |  |  | 0.0 |  | 0.0 |
| *Epilobium parviflorum* |  |  |  | 2.0 |  |  |  |  |  |  | 2.0 |
| *Eupatorium cannabium* |  |  |  |  |  |  |  | 0.0 |  |  | 0.0 |
| *Filipendula ulmaria* |  |  | 0.0 |  |  |  |  |  |  |  | 0.0 |
| *Galium aparine* | 0.0 |  |  |  | 0.0 |  |  |  |  |  | 0.0 |
| *Geranium columbinum* |  |  |  |  |  |  |  |  |  |  |  |
| *Geranium robertianum* | 9.0 |  |  |  |  |  |  | 0.0 | 1.0 |  | 10.0 |
| *Geum urbanum* | 0.3 | 0.0 |  |  |  |  |  | 0.0 | 0.0 | 0.0 | 0.3 |
| *Heracleum sphondylium* |  | 0.0 | 0.3 | 0.0 | 0.3 | 0.0 |  | 0.0 | 0.0 | 2.0 | 2.7 |
| *Hypericum tetrapterum* |  |  |  |  |  |  |  |  |  |  |  |
| *Hypochaeris radicata* |  |  |  |  | 0.7 | 0.0 | 0.7 |  |  |  | 1.3 |
| *Lamium album* |  |  |  |  |  |  |  | 0.0 |  |  | 0.0 |
| *Lantana communis* | 0.0 |  |  |  |  |  |  |  |  |  | 0.0 |
| *Lapsana communis* |  |  |  |  |  |  |  |  |  | 0.0 | 0.0 |
| *Malva sylvestris* |  |  |  |  |  |  |  |  |  |  |  |
| *Matricaria matricarioides* |  |  |  | 0.3 | 0.0 | 0.0 |  | 0.0 | 0.0 |  | 0.3 |
| *Myosoton aquaticum* |  |  |  |  |  | 0.0 |  |  |  |  | 0.0 |
| *Polygonum aviculare* |  |  |  | 0.0 |  |  |  |  |  |  | 0.0 |
| *Prunella vulgaris* |  |  |  |  |  |  | 0.7 |  |  |  | 0.7 |
| *Pulicaria dysenterica* |  |  |  |  |  |  |  |  |  |  |  |
| *Ranunculus repens* |  |  |  | 0.0 | 0.0 |  | 0.0 | 0.0 |  |  | 0.0 |
| *Rosa pimpinellifolia* |  |  | 0.0 |  |  |  |  |  |  |  | 0.0 |
| *Rubus fruticosus* | 10.9 |  | 6.0 | 10.7 | 0.0 | 1.3 | 3.0 | 1.0 | 12.0 |  | 44.9 |
| *Rubus* sp1 |  |  |  |  |  | 0.3 | 0.0 |  |  |  | 0.3 |
| *Senecio erucifolius* |  |  |  |  |  |  |  |  |  |  |  |
| *Senecio jacobaea* | 0.0 |  |  | 1.0 | 0.0 |  | 5.0 |  |  |  | 6.0 |
| *Silene dioica* |  |  | 43.0 |  |  |  |  |  |  |  | 43.0 |
| *Solanum dulcamara* |  |  |  |  |  |  |  |  |  |  |  |
| *Sonchus asper* |  |  |  |  |  |  |  |  |  |  |  |
| *Sonchus oleraceus* |  |  |  |  |  |  |  |  |  |  |  |
| *Stachys palustris* |  |  |  |  |  |  |  |  |  |  |  |
| *Stachys sylvatica* |  |  |  |  |  |  | 11.8 | 32.8 | 0.3 |  | 45.0 |
| *Stellaria media* |  |  |  |  |  |  |  |  | 0.0 |  | 0.0 |
| *Taraxacum officinale* |  |  |  |  | 0.3 | 0.0 |  |  |  |  | 0.3 |
| *Trifolium dubium* | 0.0 |  |  |  |  |  |  |  |  |  | 0.0 |
| *Trifolium pratense* |  |  |  |  |  | 0.0 | 2.0 | 0.0 |  |  | 2.0 |
| *Trifolium repens* |  |  |  |  |  |  |  |  |  |  |  |
| *Tripleurospermum inodorum* |  |  |  |  |  | 0.0 |  |  |  |  | 0.0 |
| *Vicia sepium* |  |  | 0.0 |  |  |  |  |  |  |  | 0.0 |

**Table S3. Part 2/2**

|  | **Non-invaded plots** | | | | | | | | | | **Total on non-invaded plots** |
| --- | --- | --- | --- | --- | --- | --- | --- | --- | --- | --- | --- |
| **Stigma species/ Site code** | **2** | **3** | **6** | **7** | **9** | **12** | **13** | **15** | **17** | **20** |  |
| *Achillea millefolium* |  | 0.0 |  |  |  | 1.8 |  |  |  |  | 1.8 |
| *Alliaria petiolata* |  |  |  |  |  |  |  |  |  | 0.0 | 0.0 |
| *Angelica sylvestris* |  |  |  |  |  |  |  |  |  |  |  |
| *Anthriscus sylvestris* |  |  |  |  |  |  |  |  |  |  |  |
| *Arctium minus* |  |  | 0.0 |  |  |  |  |  |  |  | 0.0 |
| *Artemisia vulgaris* |  |  |  |  |  |  |  |  |  |  |  |
| *Bellis perennis* |  |  |  |  |  | 46.3 | 0.3 | 0.0 |  |  | 46.7 |
| *Brassica* sp |  |  |  |  |  | 0.0 |  |  |  |  | 0.0 |
| *Brassica napus* |  |  |  |  |  |  |  |  |  |  |  |
| *Brassica* sp1 |  |  |  |  |  |  |  |  |  |  |  |
| *Brassica* sp2 |  |  |  |  |  |  |  |  |  | 0.0 | 0.0 |
| *Buddleja davidii* |  | 3.0 |  |  |  | 1.0 |  |  |  |  | 4.0 |
| *Calystegia sepium* | 156.0 |  |  |  |  | 9.0 | 0.0 | 0.0 | 402.0 |  | 567.0 |
| *Capsella bursa-pastoris* |  |  |  |  |  | 0.0 |  |  |  |  | 0.0 |
| *Centaurea nigra* |  | 16.3 |  |  | 0.0 |  |  |  |  |  | 16.3 |
| *Chamerion angustifolium* |  |  |  |  |  |  |  |  |  |  |  |
| *Circaea lutetiana* |  |  | 0.0 |  |  |  |  |  |  |  | 0.0 |
| *Cirsium arvense* | 0.0 | 0.0 |  |  | 0.3 |  | 1.0 | 0.0 | 4.3 |  | 5.7 |
| *Cirsium palustre* |  |  |  | 16.3 |  |  |  |  |  |  | 16.3 |
| *Cirsium vulgare* | 4.0 |  |  |  |  | 0.7 |  |  |  |  | 4.7 |
| *Clematis vitalba* |  | 0.0 |  |  |  | 49.8 | 0.3 | 0.0 |  |  | 50.2 |
| *Convolvulus arvensis* |  |  |  |  |  |  |  |  | 1.0 |  | 1.0 |
| *Dipsacus fullonum* |  | 0.7 |  |  |  |  |  |  |  |  | 0.7 |
| *Dipsacus pilosus* |  |  |  |  |  |  |  |  |  |  |  |
| *Epilobium hirsutum* | 1.3 | 4.3 | 0.7 |  | 131.7 |  | 2.3 | 0.0 | 25.5 |  | 165.8 |
| *Epilobium montanum* |  |  |  |  |  |  |  |  |  |  |  |
| *Epilobium parviflorum* |  |  | 0.3 |  |  |  |  |  |  |  | 0.3 |
| *Eupatorium cannabium* |  | 0.3 |  |  |  |  |  |  |  |  | 0.3 |
| *Filipendula ulmaria* |  |  |  |  |  |  |  |  |  |  |  |
| *Galium aparine* |  |  |  |  |  |  |  | 0.0 |  |  | 0.0 |
| *Geranium columbinum* |  |  |  |  |  | 0.3 |  |  |  |  | 0.3 |
| *Geranium robertianum* |  |  | 0.0 |  |  |  |  |  |  |  | 0.0 |
| *Geum urbanum* |  |  |  |  |  |  |  | 0.0 |  | 0.0 | 0.0 |
| *Heracleum sphondylium* | 12.7 |  | 0.0 | 0.0 |  |  | 0.0 |  |  |  | 12.7 |
| *Hypericum tetrapterum* |  |  | 2.7 |  |  |  |  |  |  |  | 2.7 |
| *Hypochaeris radicata* |  | 9.7 |  |  |  | 2.3 | 0.0 |  | 0.0 |  | 12.0 |
| *Lamium album* |  |  |  |  |  | 0.3 |  | 0.0 |  |  | 0.3 |
| *Lantana communis* |  |  |  |  |  |  |  |  |  |  |  |
| *Lapsana communis* |  |  |  |  |  |  |  |  |  | 0.0 | 0.0 |
| *Malva sylvestris* |  |  |  |  |  |  |  |  |  | 41.3 | 41.3 |
| *Matricaria matricarioides* |  |  |  |  |  |  |  |  |  |  |  |
| *Myosoton aquaticum* |  |  |  |  |  |  |  |  |  |  |  |
| *Polygonum aviculare* |  |  |  |  |  |  |  |  |  |  |  |
| *Prunella vulgaris* |  |  |  |  |  |  | 0.0 | 0.0 |  |  | 0.0 |
| *Pulicaria dysenterica* |  |  |  |  | 0.3 |  |  |  |  |  | 0.3 |
| *Ranunculus repens* | 0.7 |  |  | 0.0 |  | 5.7 | 0.0 | 0.0 |  |  | 6.3 |
| *Rosa pimpinellifolia* |  |  |  |  |  |  |  |  |  |  |  |
| *Rubus fruticosus* | 0.0 | 0.0 | 0.0 |  | 0.0 | 0.0 | 0.0 | 0.0 | 0.3 |  | 0.3 |
| *Rubus* sp1 |  |  |  |  |  |  | 0.0 | 0.0 |  |  | 0.0 |
| *Senecio erucifolius* |  |  |  |  |  | 0.7 |  |  |  |  | 0.7 |
| *Senecio jacobaea* |  | 7.0 |  |  | 0.0 |  |  |  |  |  | 7.0 |
| *Silene dioica* |  |  |  |  |  |  |  |  |  |  |  |
| *Solanum dulcamara* |  |  |  |  |  |  |  | 0.0 |  |  | 0.0 |
| *Sonchus asper* |  |  |  |  |  | 2.3 |  |  |  |  | 2.3 |
| *Sonchus oleraceus* |  |  |  |  |  |  |  |  |  | 0.0 | 0.0 |
| *Stachys palustris* | 2.5 |  |  |  |  |  |  |  |  |  | 2.5 |
| *Stachys sylvatica* |  |  | 0.0 |  |  | 0.0 | 0.0 | 0.0 |  |  | 0.0 |
| *Stellaria media* |  |  |  |  |  |  |  |  |  |  |  |
| *Taraxacum officinale* |  |  |  |  |  | 31.0 | 0.0 | 0.0 |  | 0.0 | 31.0 |
| *Trifolium dubium* |  |  | 0.0 |  |  |  |  |  |  |  | 0.0 |
| *Trifolium pratense* | 2.0 | 0.0 | 0.0 | 0.0 | 0.0 | 0.7 | 0.0 | 0.3 |  |  | 3.0 |
| *Trifolium repens* |  | 0.0 | 0.0 |  | 0.0 |  | 0.0 | 0.0 | 0.0 |  | 0.0 |
| *Tripleurospermum inodorum* |  |  |  |  |  |  |  |  |  |  |  |
| *Vicia sepium* |  |  |  |  |  |  |  |  |  |  |  |
