# Supplementary material for: The Impact of the Invasive Alien Plant, Impatiens glandulifera, on Pollen Transfer Networks
Source: PLoS One. 2015 Dec 3;10(12):e0143532. doi: 10.1371/journal.pone.0143532 (PMC4669169; doi:10.1371/journal.pone.0143532)
Supplement: S4 Table — Full model: y = meanpollen ~ habitat*stigmatype + (1|sitecode/stigmaspecies), family = Gamma (link = log) (DOCX) [file pone.0143532.s004.docx]

**S4 Table.** **Results of the Generalized Linear Mixed Models (GLMM`s) and Post-hoc tests (Tukey) testing whether the deposition of balsam (*Impatiens glandulifera*), conspecific and heterospecific pollen grains on stigmas are different between invaded and non-invaded habitats, and whether it is affected by the stigma type (dry, semidry, wet)**. Full model: y = mean pollen ~ habitat*stigma type + (1|site code/stigma species), family=Gamma (link=log).

|  | **Balsam** | | **Conspecific** | | **Heterospecific** | |  |
| --- | --- | --- | --- | --- | --- | --- | --- |
| **GLMM`s** | **Est** | ***p*** | **Est** | ***p*** | **Est** | ***p*** |  |
| Intercept (habitat invaded, stigma dry) | 2.382 | 0.000 | 4.158 | 0.000 | 1.397 | 0.000 |  |
| Habitat –non invaded | -1.827 | 0.004 | -0.069 | 0.780 | 0.267 | 0.583 |  |
| Stigma - semidry | -1.753 | 0.021 | -0.369 | 0.242 | -0.768 | 0.176 |  |
| Stigma - wet | -1.721 | 0.054 | -0.522 | 0.128 | -0.459 | 0.470 |  |
| Habitat non-invaded*stigma semidry | 2.077 | 0.069 | 0.630 | 0.163 | 0.352 | 0.671 |  |
| Habitat non-invaded*stigma wet | 1.312 | 0.373 | 0.685 | 0.156 | 0.967 | 0.363 |  |
| **Post-hoc test** |  |  |  |  |  |  |  |
| Non-invaded dry - invaded dry | -1.827 | 0.047 | - | - | - | - |  |
| Invaded semidry – invaded dry | -1.753 | 0.178 | - | - | - | - |  |
| Non-invaded semidry – invaded dry | -1.502 | 0.415 | - | - | - | - |  |
| Invaded wet - invaded dry | -1.719 | 0.374 | - | - | - | - |  |
| Non-invaded wet – invaded dry | -2.229 | 0.351 | - | - | - | - |  |
| Invaded semidry – non-invaded dry | 0.074 | 1 | - | - | - | - |  |
| Non-invaded semidry – non-invaded dry | 0.325 | 0.999 | - | - | - | - |  |
| Invaded wet - non-invaded dry | 0.108 | 1 | - | - | - | - |  |
| Non-invaded wet – non-invaded dry | -0.403 | 0.999 | - | - | - | - |  |
| Non-invaded semidry - invaded semidry | 0.251 | 0.999 | - | - | - | - |  |
| Invaded wet – invaded semidry | 0.034 | 1 | - | - | - | - |  |
| Non-invaded wet – invaded semidry | -0.477 | 0.999 | - | - | - | - |  |
| Invaded wet – non-invaded semidry | -0.217 | 0.999 | - | - | - | - |  |
| Non – invaded wet - non-invaded semidry | -0.728 | 0.992 | - | - | - | - |  |
| Non-invaded wet – invaded wet | -0.510 | 0.999 | - | - | - | - |  |
