# Supplementary material for: The Impact of the Invasive Alien Plant, Impatiens glandulifera, on Pollen Transfer Networks
Source: PLoS One. 2015 Dec 3;10(12):e0143532. doi: 10.1371/journal.pone.0143532 (PMC4669169; doi:10.1371/journal.pone.0143532)
Supplement: S5 Table — The mean is the total number of pollen grains counted on the stigmas of each species divided by the total number of stigmas sampled, with data pooled from the 20 sites. (DOCX) [file pone.0143532.s005.docx]

**S5 Table**. **The five species with the highest mean deposition of pollen grains per stigma, according to each type of pollen: conspecific, balsam and heterospecific.** The mean is the total number of pollen grains counted on the stigmas of each species divided by the total number of stigmas that contain the respective pollen type, with data pooled from the 20 sites.

|  | **Conspecific** | | | **Balsam** | | | **Heterospecific** | | |
| --- | --- | --- | --- | --- | --- | --- | --- | --- | --- |
|  | **Mean** | **Total** | **Stigmas** | **Mean** | **Total** | **Stigmas** | **Mean** | **Total** | **Stigmas** |
| *Vicia sepium* | 2302.00 | 6906 | 3 | - | - | - | - | - | - |
| *Hypericum tetrapterum* | 575.26 | 24161 | 42 | - | - | - | - | - | - |
| *Buddleja davidii* | 364.72 | 13191 | 36 | - | - | - | - | - | - |
| *Epilobium montanum* | 420.50 | 3364 | 8 | - | - | - | - | - | - |
| *Hypochaeris radicata* | 364.72 | 24801 | 68 | - | - | - | - | - | - |
| *Calystegia sepium* | - | - | - | 277.9 | 16952 | 61 | 78.42 | 4784 | 61 |
| *Chamerion angustifolium* | - | - | - | 91.64 | 1283 | 14 |  |  |  |
| *Silene dioica* | - | - | - | 21.50 | 43 | 2 | 63.50 | 127 | 2 |
| *Epilobium hirsutum* | - | - | - | 10.98 | 7225 | 658 | - | - | - |
| *Circaea lutetiana* | - | - | - | 9.38 | 2777 | 296 | - | - | - |
| *Angelica sylvestris* | - | - | - | - | - | - | 298.78 | 2689 | 9 |
| *Cirsium vulgare* | - | - | - | - | - | - | 69.97 | 2099 | 30 |
| *Brassica* sp1 | - | - | - | - | - | - | 67.00 | 201 | 3 |
