# Supplementary material for: The Impact of the Invasive Alien Plant, Impatiens glandulifera, on Pollen Transfer Networks
Source: PLoS One. 2015 Dec 3;10(12):e0143532. doi: 10.1371/journal.pone.0143532 (PMC4669169; doi:10.1371/journal.pone.0143532)
Supplement: S6 Table — Values in bold are significant results at p < 0.05. The results show the significant p values for each term in the full model which has habitat (phabitat) as the main effect and stigma (pstigmasp) and pollen species richness (ppollensp) as covariates. § Results of a Kruskal-Wallis test fitted to the non-normal H`2 data; each p-value correspondent to an independent non-parametric Kruskal-Wallis test. (DOCX) [file pone.0143532.s006.docx]

**S6 Table**. **Results of the Analysis of Variance testing the effects of the presence of balsam (*Impatiens glandulifera*) on the structure of pollen transfer networks**. Values in bold are significant results at p < 0.05. The results show the significant p values for each term in the full model which has habitat (p_habitat_) as the main effect and stigma (p_stigmasp_) and pollen species richness (p_pollensp_) as covariates. ^§^ Results of a Kruskal-Wallis test fitted to the non-normal H`2 data; each p-value correspondent to an independent non-parametric Kruskal-Wallis test.

|  | p_habitat_ | p_stigmasp_ | p_pollensp_ | p_total_ |
| --- | --- | --- | --- | --- |
| Connectance (log[wc +1]) | 0.27 | **0.002** | **0.03** | **4.36e-05** |
| Nestedness (log[wnodf +1)] | 0.07 | **0.0001** | **0.04** | **0.001** |
| Interaction evenness | 0.87 | 0.53 | 0.61 | 0.93 |
| Number of interactions | 0.38 | 0.54 | **0.002** | **0.008** |
| Modularity | 0.24 | **0.03** | 0.62 | 0.09 |
| Linkage density (log[ld]) | 0.24 | 0.09 | 0.35 | 0.32 |
| H`2^§^ | 0.30 | 0.63 | 0.57 | 0.30 |
