# Supplementary material for: The Impact of the Invasive Alien Plant, Impatiens glandulifera, on Pollen Transfer Networks
Source: PLoS One. 2015 Dec 3;10(12):e0143532. doi: 10.1371/journal.pone.0143532 (PMC4669169; doi:10.1371/journal.pone.0143532)
Supplement: S1 Fig — Red points are areas invaded by balsam (Impatiens glandulifera); blue areas are areas non-invaded by balsam, and used as “control” plots. Figure created using OpenStreetMap for illustrative purposes only. Data is available under the Open Database License. (DOCX) [file pone.0143532.s007.docx]

**S1 Fig.** **Map of the study area around the city of Bristol, UK**. Red points are areas invaded by balsam (*Impatiens glandulifera)*; blue areas are adjacent areas non-invaded by balsam, and used as control plots. Figure created using OpenStreetMap for illustrative purposes only. Data is available under the Open Database License.

**
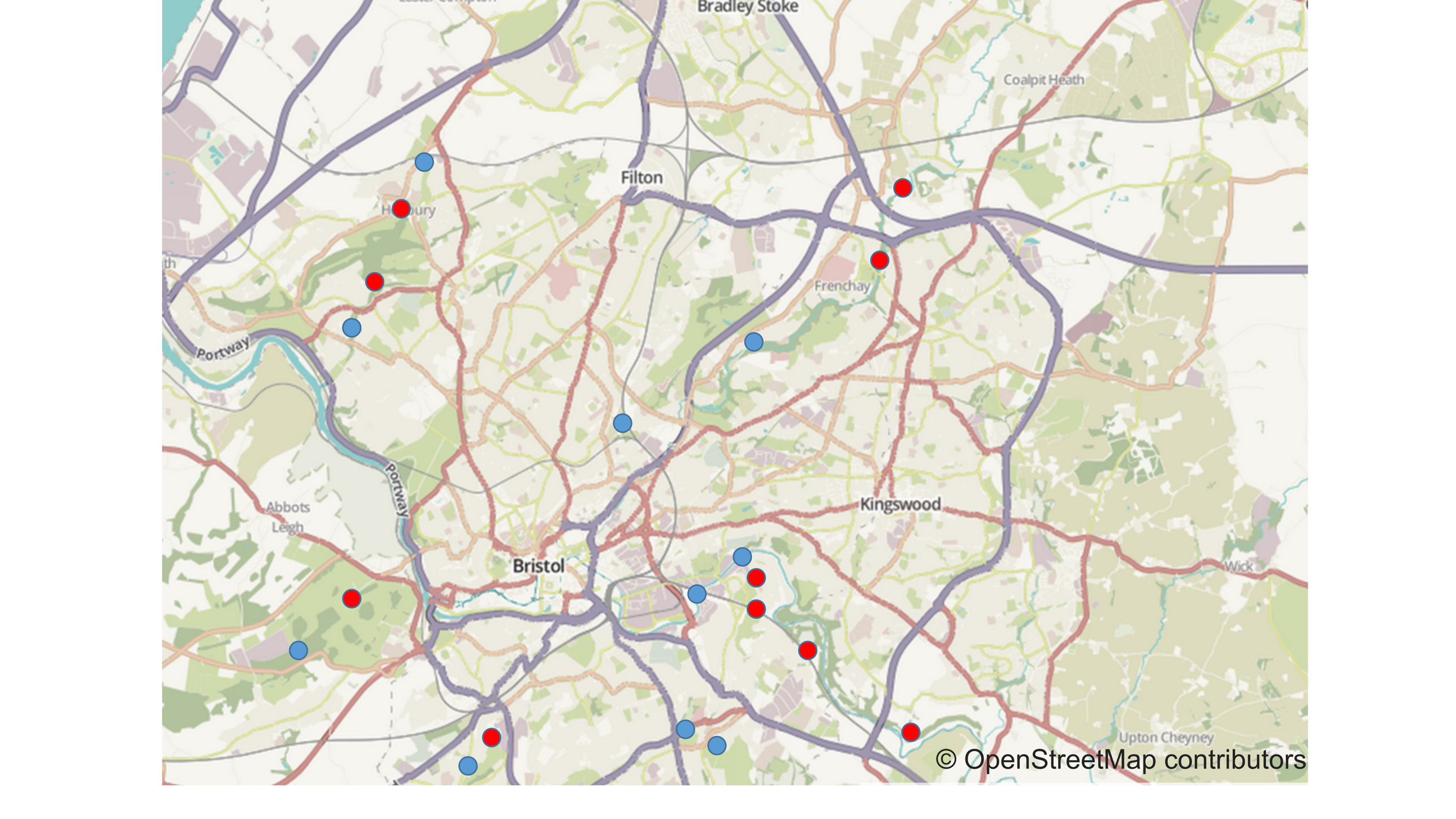
**
