# Supplementary material for: The Impact of the Invasive Alien Plant, Impatiens glandulifera, on Pollen Transfer Networks
Source: PLoS One. 2015 Dec 3;10(12):e0143532. doi: 10.1371/journal.pone.0143532 (PMC4669169; doi:10.1371/journal.pone.0143532)
Supplement: S2 Fig — Upper panels show the distribution of frequencies of the number of balsam pollen grains per sample in each habitat. Lower panels show the distribution of balsam pollen grains per sample across the different sites in each habitat. (DOCX) [file pone.0143532.s008.docx]

**
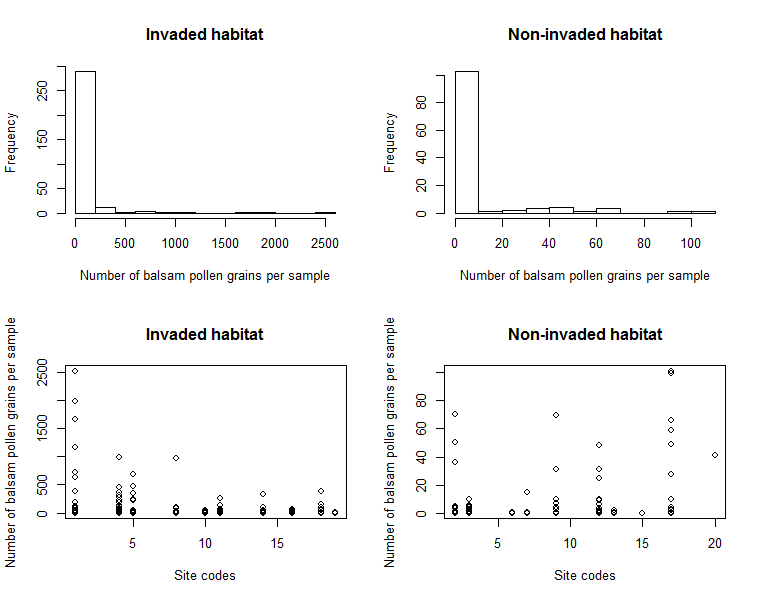
S2 FIg**. **Distribution of balsam (*Impatiens glandulifera*) pollen grains found in stigmas of plants occurring in invaded and non-invaded habitats**. Upper panels show the distribution of frequencies of the number of balsam pollen grains per sample in each habitat (sites pooled). Lower panels show the distribution of balsam pollen grains per sample across the different sites in each habitat.
