# Supplementary material for: The Impact of the Invasive Alien Plant, Impatiens glandulifera, on Pollen Transfer Networks
Source: PLoS One. 2015 Dec 3;10(12):e0143532. doi: 10.1371/journal.pone.0143532 (PMC4669169; doi:10.1371/journal.pone.0143532)

**S3 Fig**. **Frequency histograms showing the number of pollen grains per stigma species; the pollen grains are classified as (a) conspecific, (b) balsam (*Impatiens glandulifera*) and (c) heterospecific (pollen grains of all other species found in the 20 study sites).**


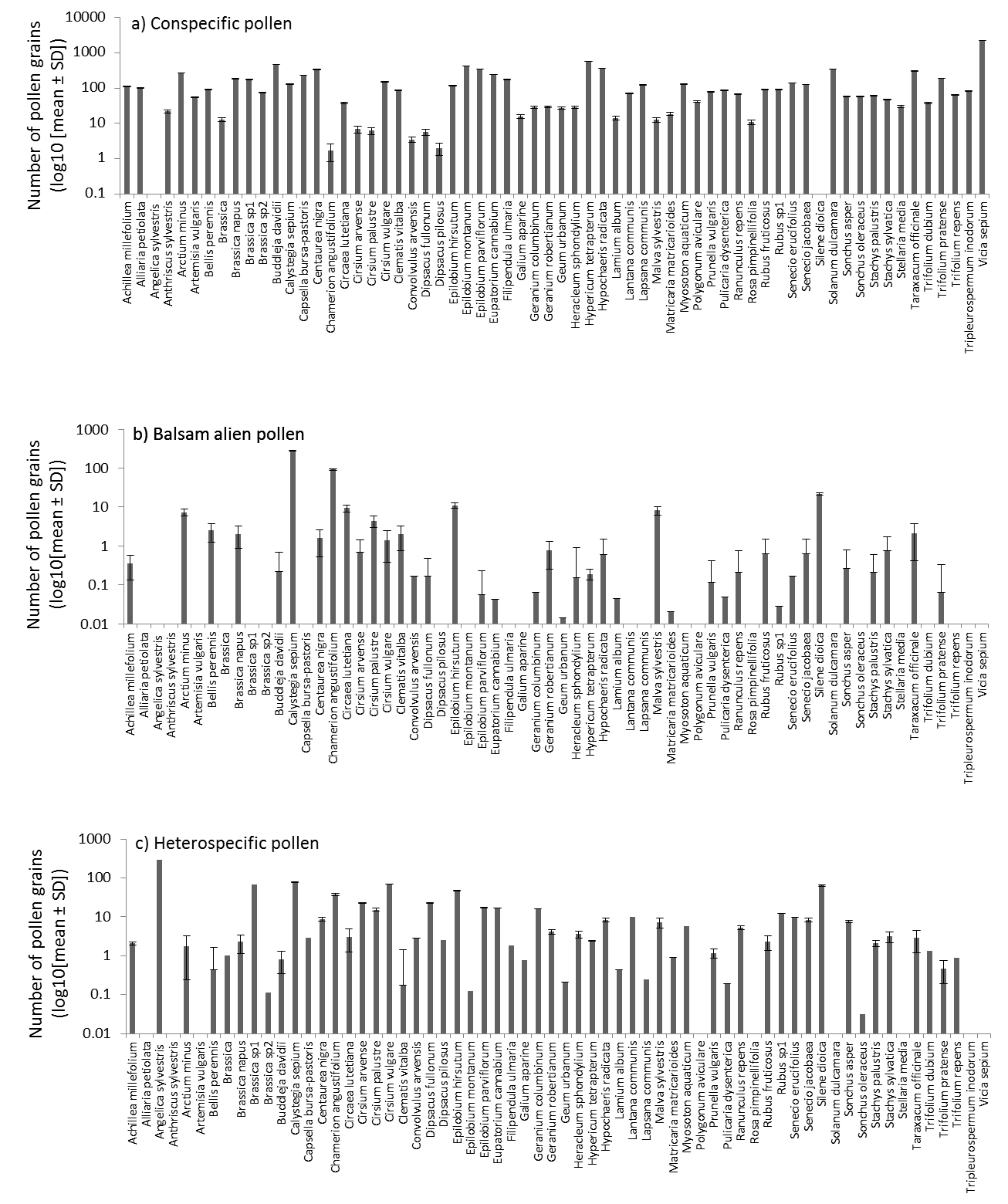

Supplement: S3 Fig — (DOCX) [file pone.0143532.s009.docx]
