# Supplementary material for: The Impact of the Invasive Alien Plant, Impatiens glandulifera, on Pollen Transfer Networks
Source: PLoS One. 2015 Dec 3;10(12):e0143532. doi: 10.1371/journal.pone.0143532 (PMC4669169; doi:10.1371/journal.pone.0143532)

**S4 Fig. Pollen transfer networks invaded and non-invaded by balsam, *Impatiens glandulifera* Royle.** Networks are shown in pairs, in the order the data was collected. Top species are pollen grains; bottom species are stigma species. The width of the rectangles in top and bottom side of the network and the width of the triangles linking both sides represent the abundance of each species and the frequency of interactions, respectively. Red interactions are those with *I. glandulifera*.


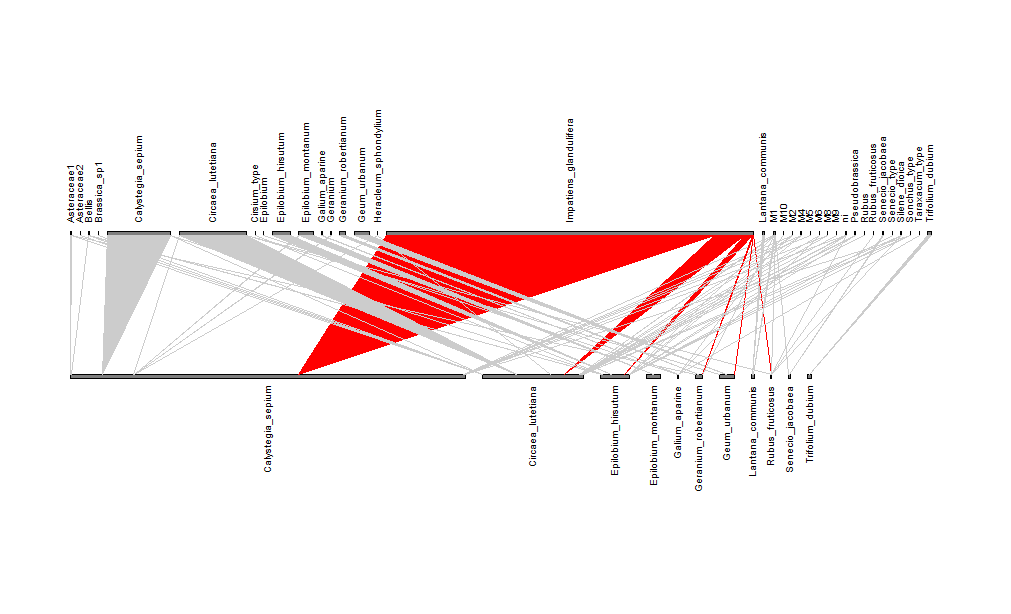
Site 1 – INVADED

Site 2 – NON-INVADED


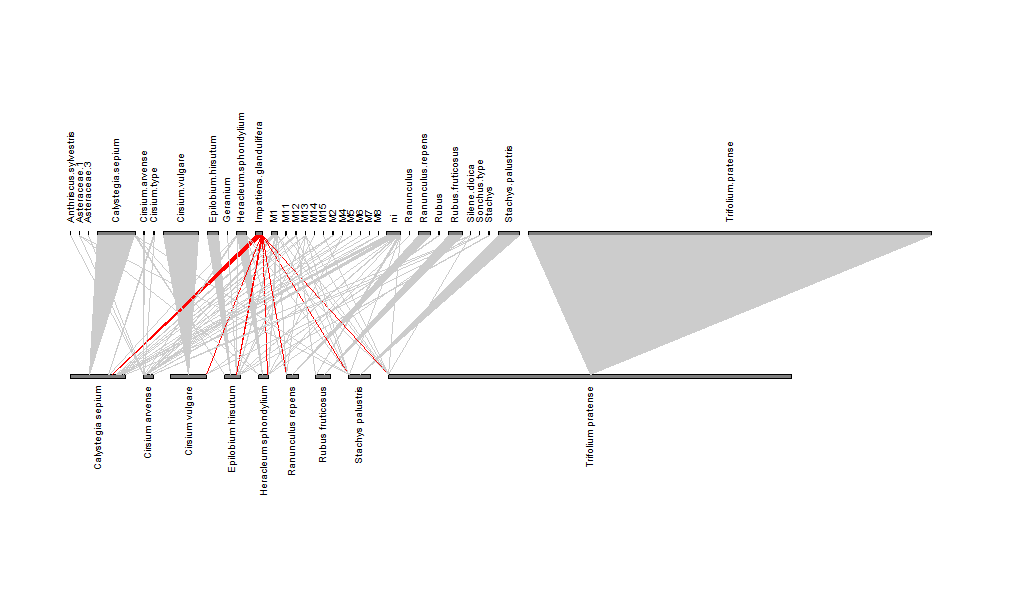
Site 3 – NON-INVADED


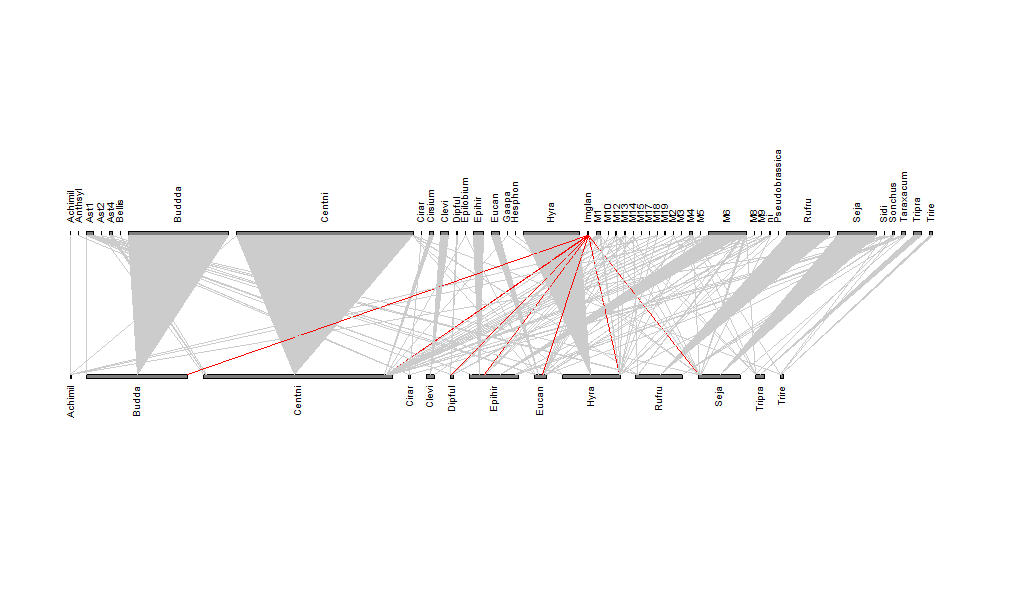


Site 4 - INVADED


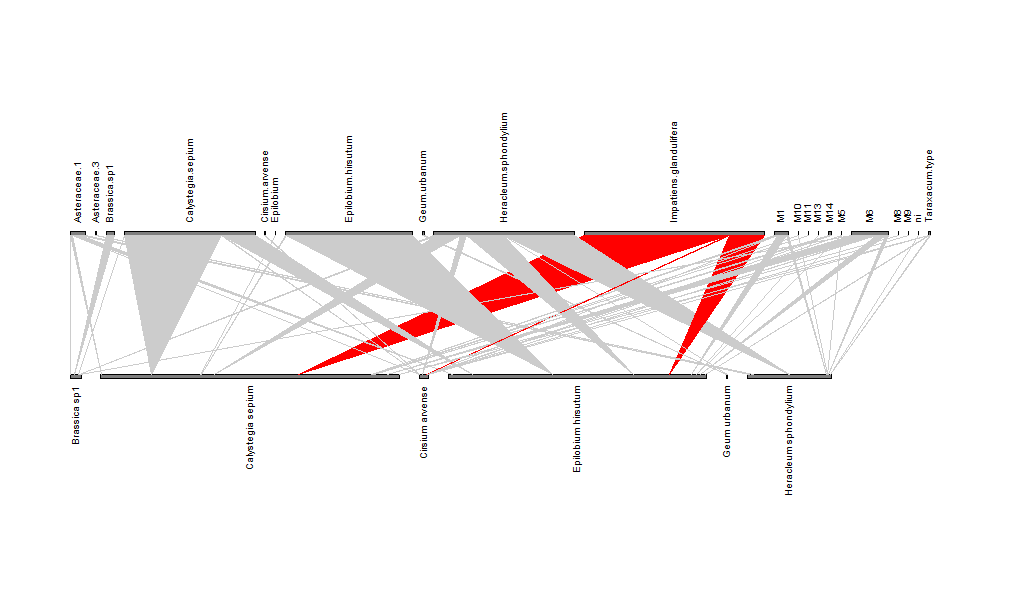


Site5 - INVADED


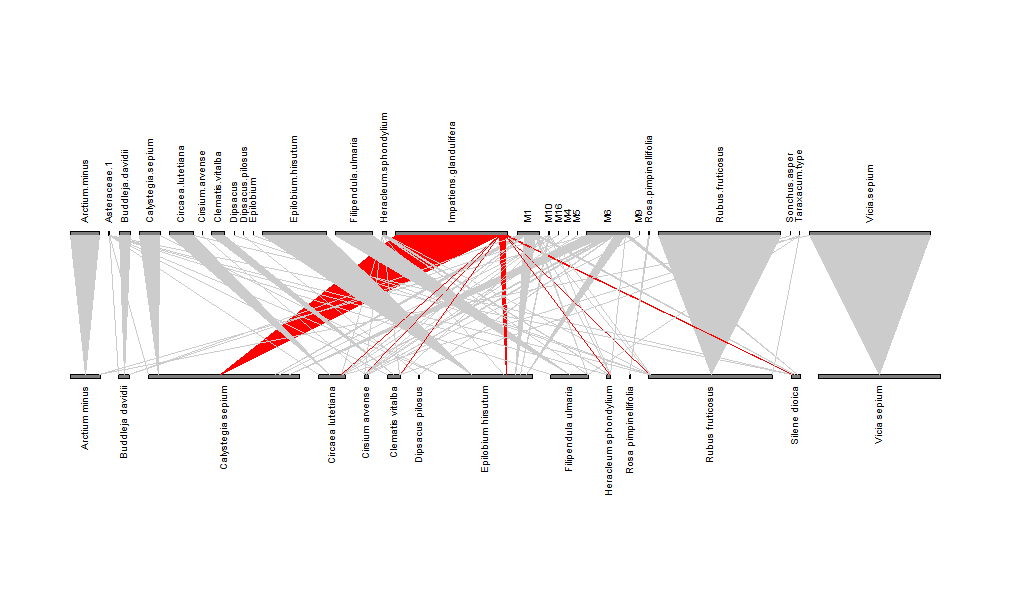


Site 6 – NON-INVADED


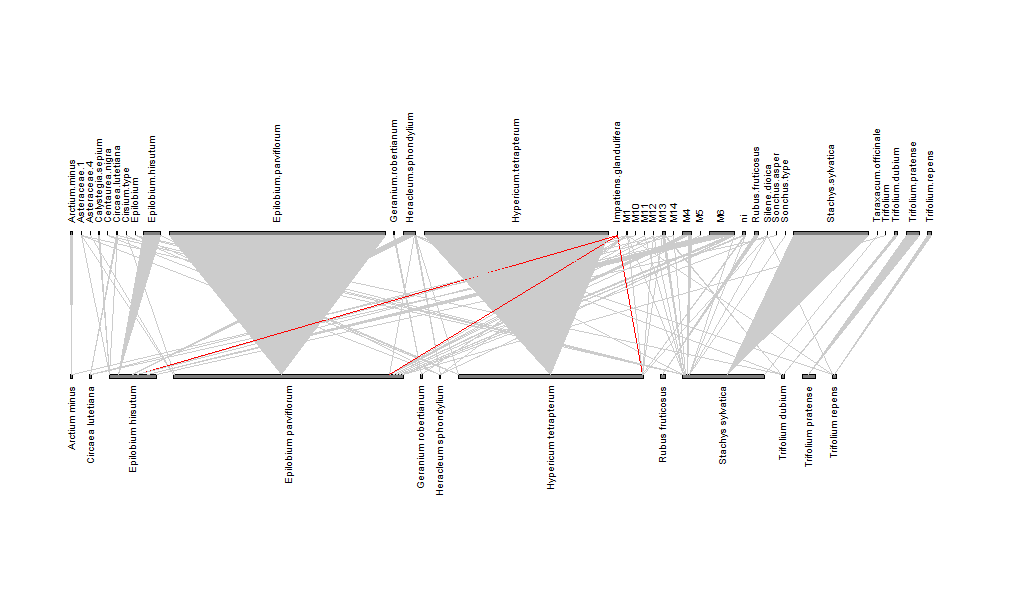


Site 7 – NON-INVADED


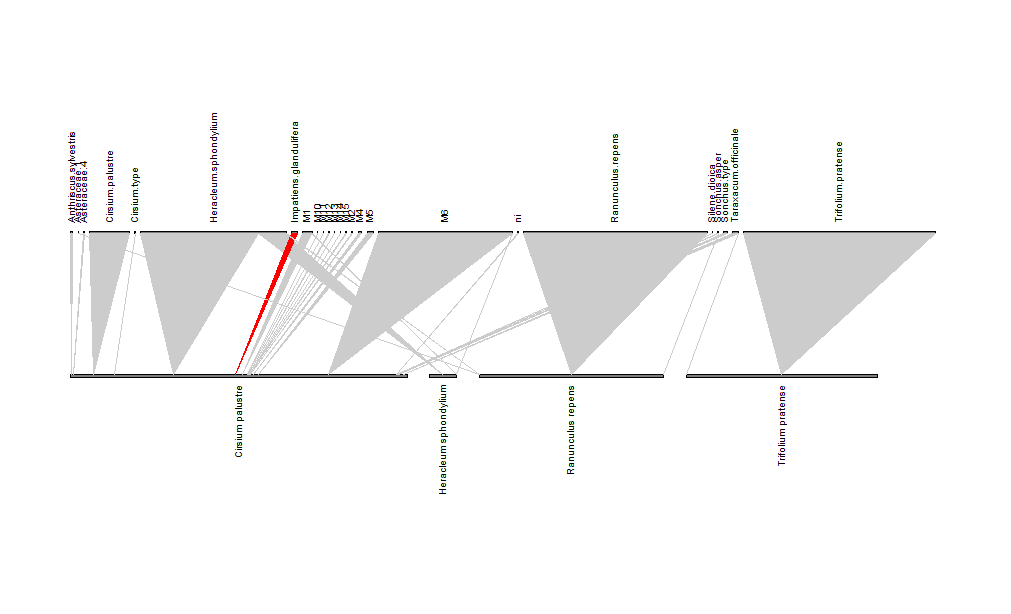


Site 8 - INVADED


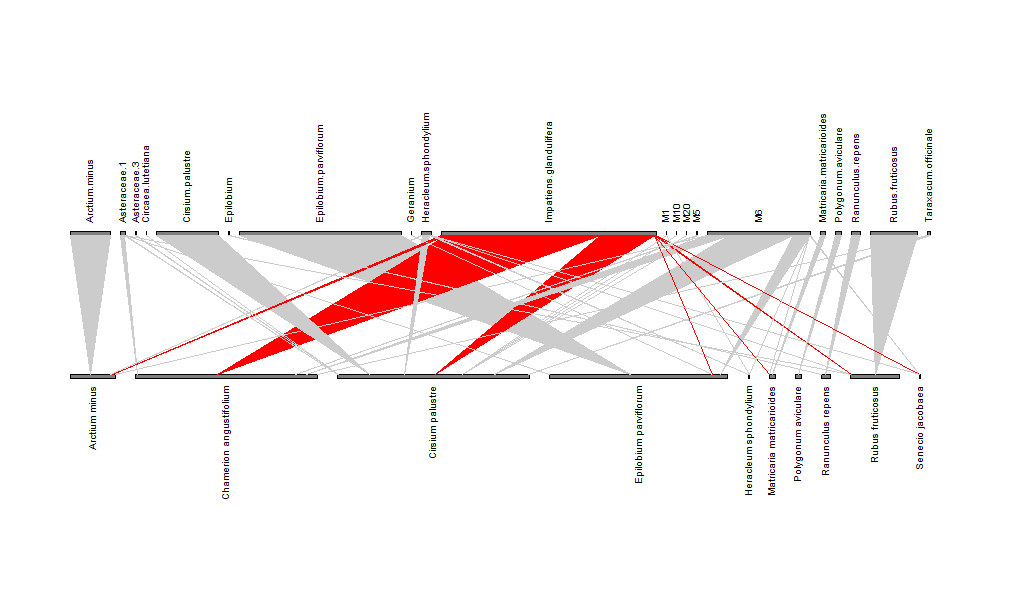


Site 9 – NON-INVADED


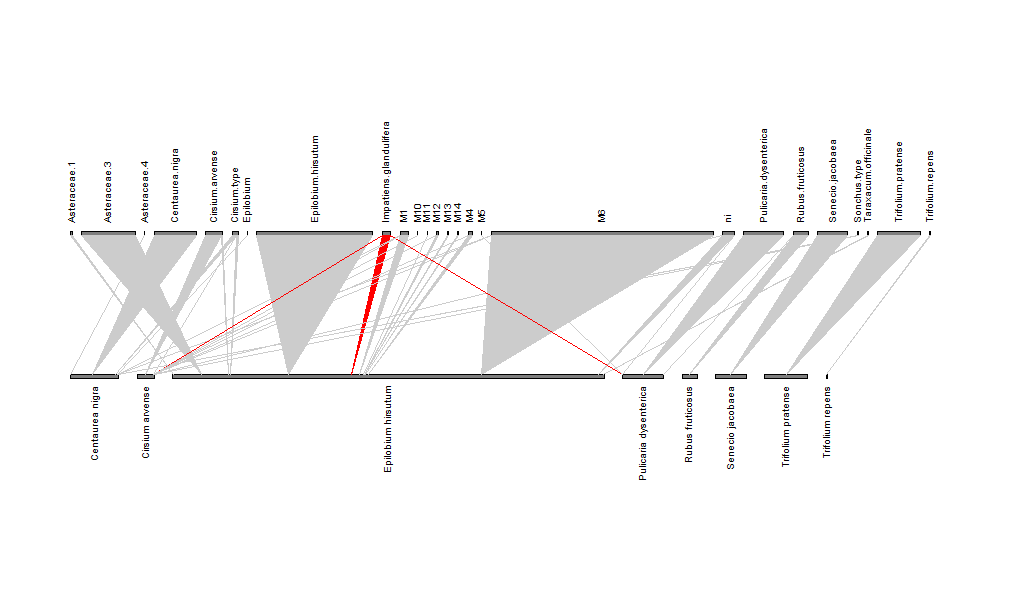


Site 10 - INVADED


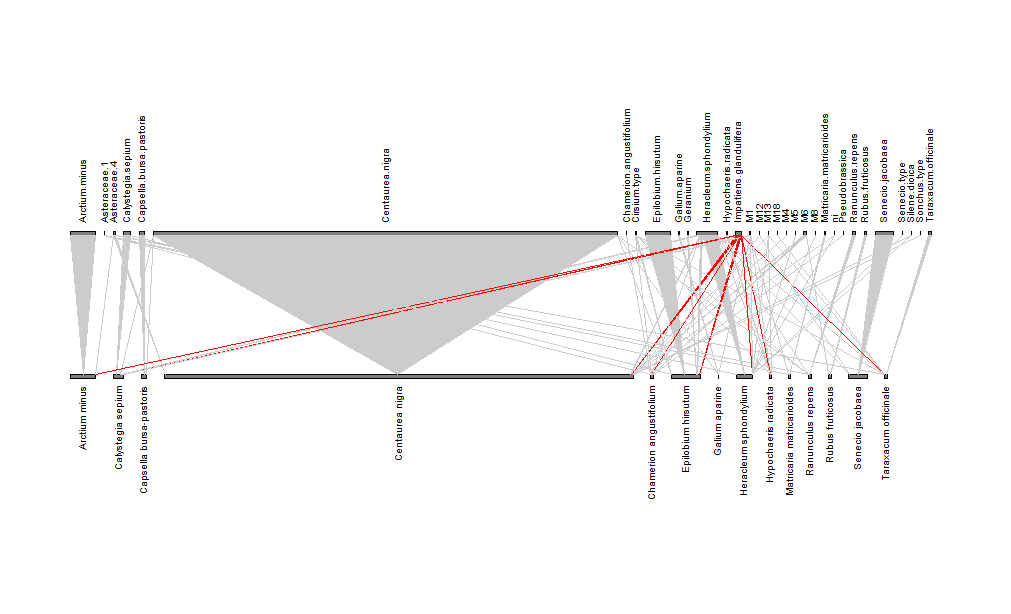


Site 11 - INVADED


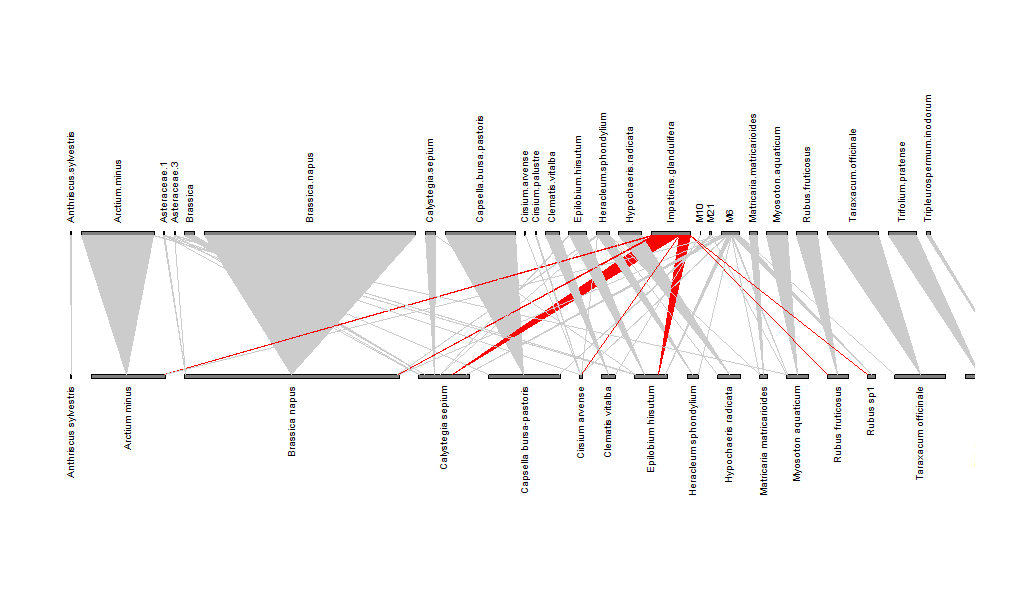


Site 12 – NON-INVADED


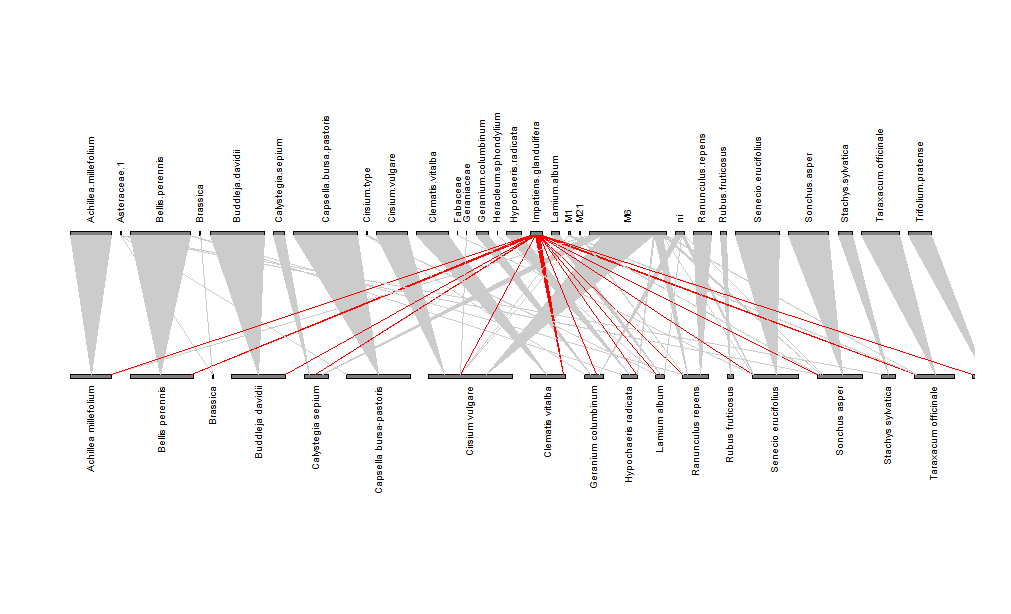


Site 13 – NON-INVADED


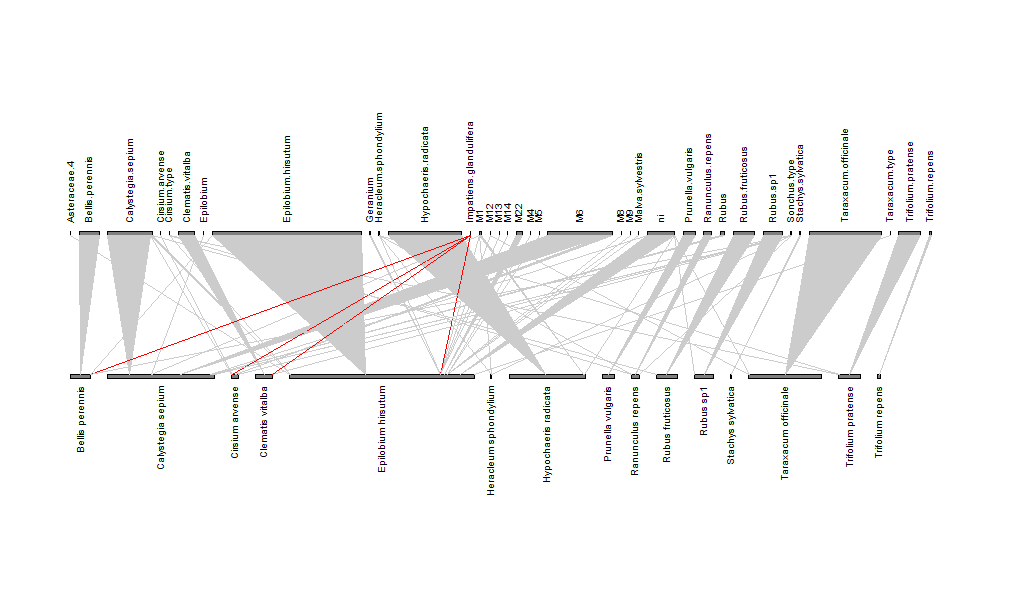


Site 14 - INVADED


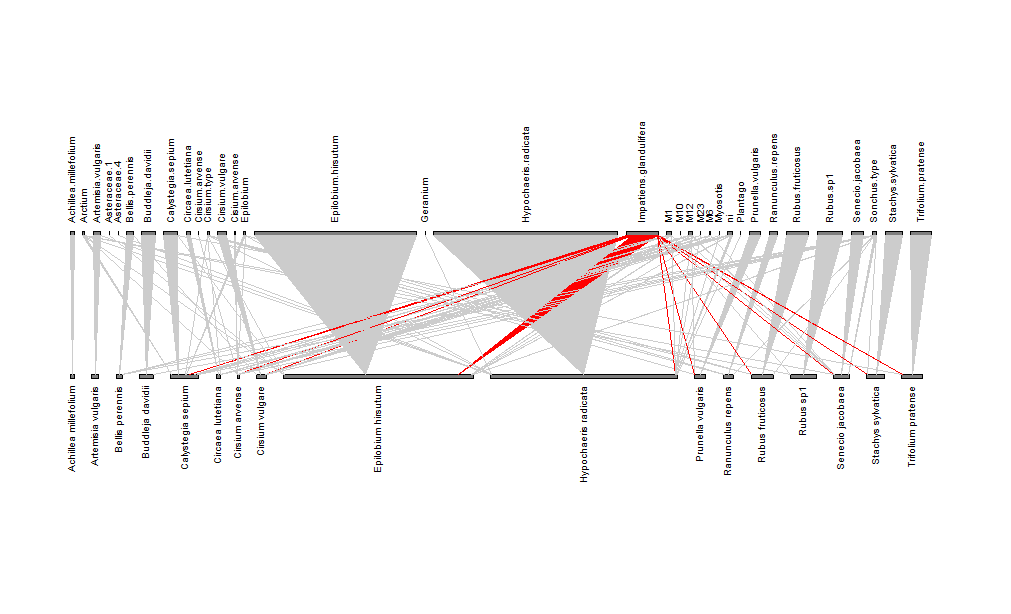


Site 15 – NON-INVADED


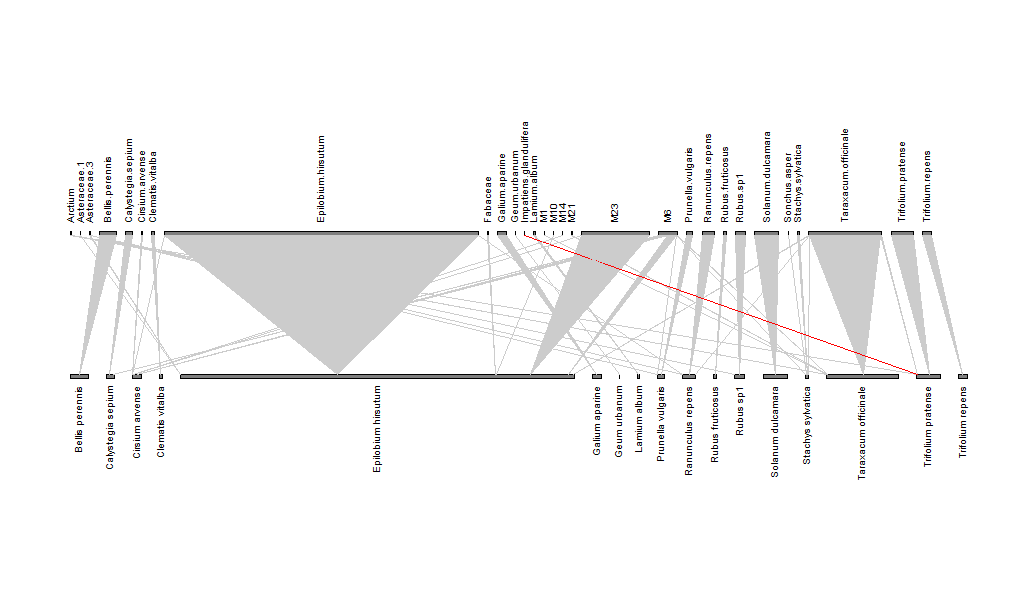


Site 16 - INVADED


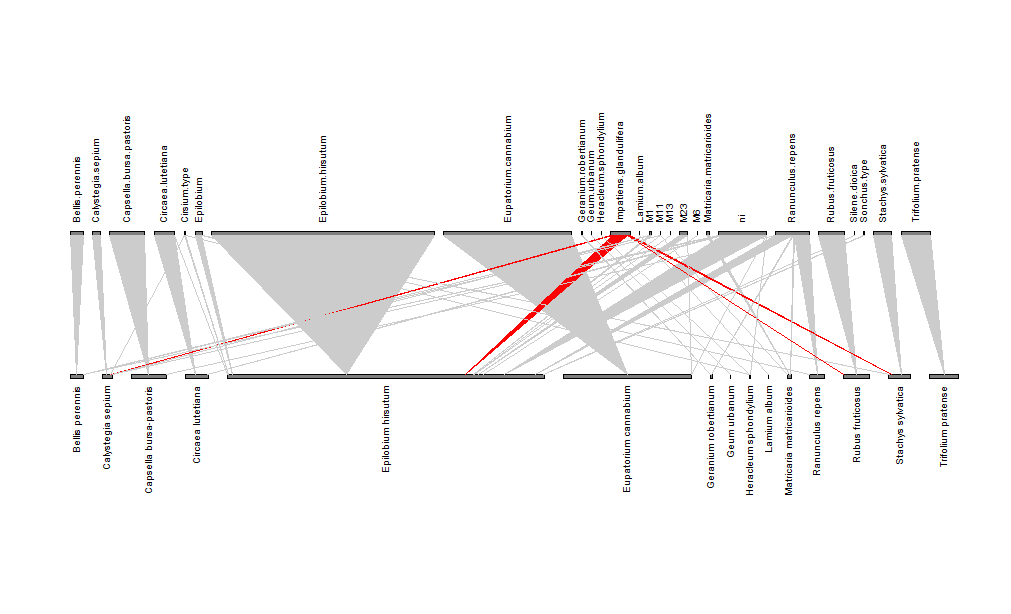


Site 17 – NON-INVADED


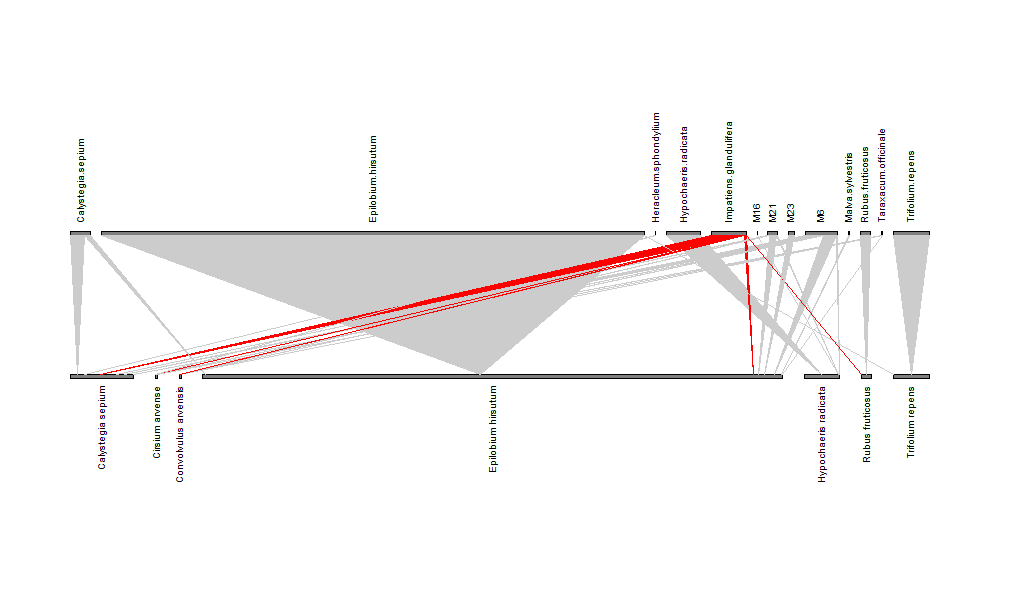


Site 18 - INVADED


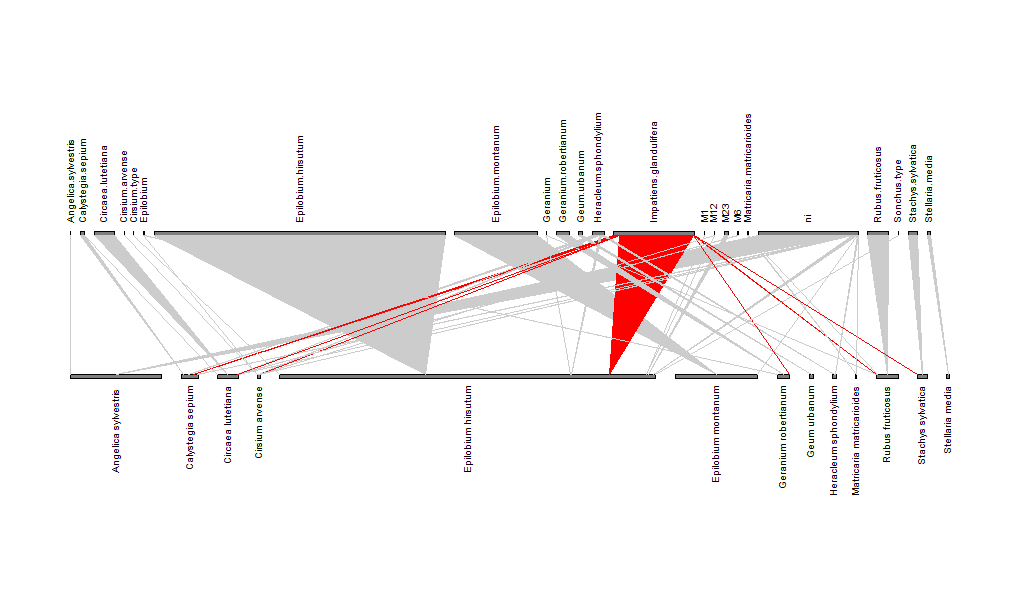


Site 19 - INVADED


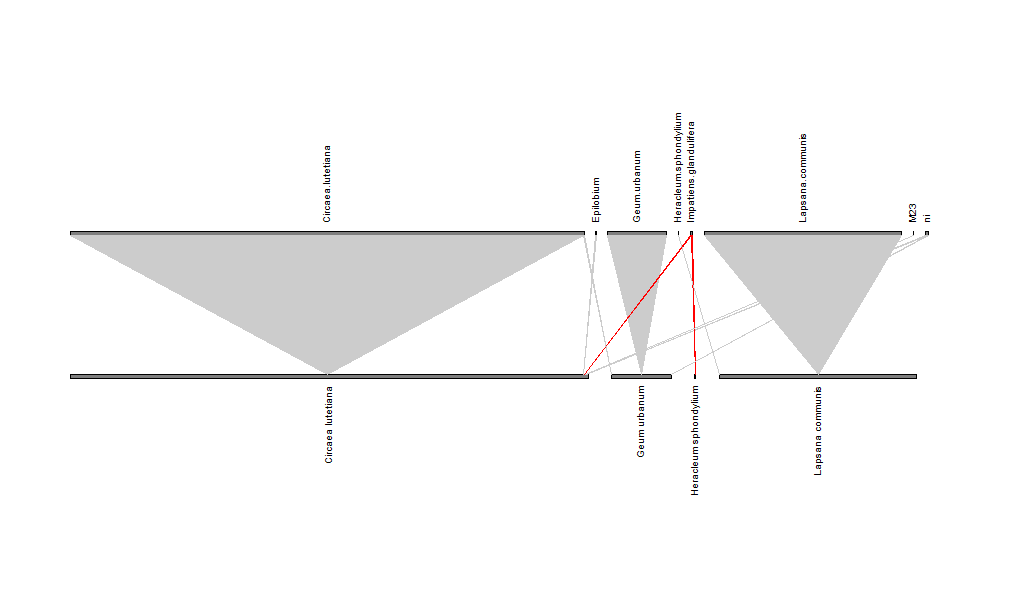


Site 20 – NON-INVADED


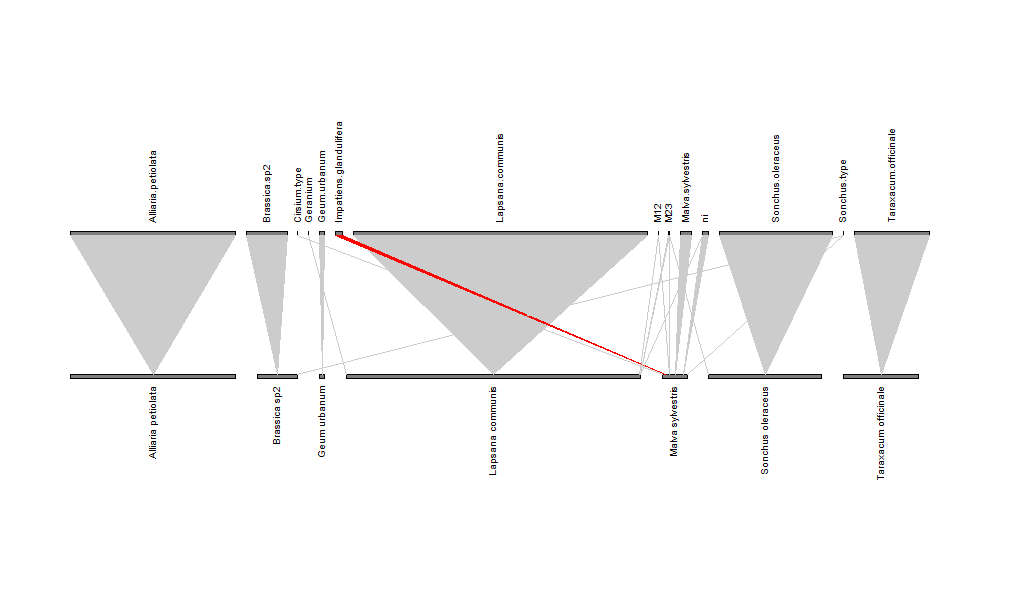

Supplement: S4 Fig — Networks are shown in pairs, in the order the data was collected. Top species are pollen grains; bottom species are stigma species. The width of the rectangles in top and bottom side of the network and the width of the triangles linking both sides represent the abundance of each species and the frequency of interactions, respectively. Red interactions are those with I. glandulifera. (DOCX) [file pone.0143532.s010.docx]
